# Nanoparticles Dysregulate the Human Placental Secretome with Consequences on Angiogenesis and Vascularization

**DOI:** 10.1002/advs.202401060

**Published:** 2024-05-20

**Authors:** Battuja Dugershaw‐Kurzer, Jonas Bossart, Marija Buljan, Yvette Hannig, Sarah Zehnder, Govind Gupta, Vera M. Kissling, Patrycja Nowak‐Sliwinska, Judy R. van Beijnum, Arjan W. Griffioen, Stefan Masjosthusmann, Etta Zühr, Ellen Fritsche, René Hornung, Thomas Rduch, Tina Buerki‐Thurnherr

**Affiliations:** ^1^ Laboratory for Particles‐Biology Interactions Swiss Federal Laboratories for Materials Science and Technology (Empa) St. Gallen 9014 Switzerland; ^2^ Department of Health Sciences and Technology ETH Zurich Zurich 8093 Switzerland; ^3^ SIB Swiss Institute of Bioinformatics Lausanne 1015 Switzerland; ^4^ Institute of Pharmaceutical Sciences of Western Switzerland Geneva 1211 Switzerland; ^5^ School of Pharmaceutical Sciences University of Geneva Geneva 1205 Switzerland; ^6^ Angiogenesis Laboratory Department of Medical Oncology UMC loacation Vrije Universiteit Amsterdam Amsterdam 1081 The Netherlands; ^7^ IUF—Leibniz Research Institute for Environmental Medicine 40225 Duesseldorf Germany; ^8^ Medical Faculty Heinrich Heine University 40225 Duesseldorf Germany; ^9^ DNTOX GmbH 40223 Duesseldorf Germany; ^10^ Department of Gynaecology and Obstetrics Cantonal Hospital St.Gallen (KSSG) St. Gallen 9007 Switzerland

**Keywords:** angiogenesis, developmental toxicity, nanoparticles, placenta, secretome

## Abstract

Exposure to nanoparticles (NPs) in pregnancy is increasingly linked to adverse effects on embryo‐fetal development and health later in life. However, the developmental toxicity mechanisms of NPs are largely unknown, in particular potential effects on the placental secretome, which orchestrates many developmental processes pivotal for pregnancy success. This study demonstrates extensive material‐ and pregnancy stage‐specific deregulation of placental signaling from a single exposure of human placental explants to physiologically relevant concentrations of engineered (silica (SiO_2_) and titanium dioxide (TiO_2_) NPs) and environmental NPs (diesel exhaust particles, DEPs). This includes a multitude of secreted inflammatory, vascular, and endocrine placental factors as well as extracellular vesicle (EV)‐associated proteins. Moreover, conditioned media (CM) from NP‐exposed explants induce pronounced anti‐angiogenic and anti‐vasculogenic effects, while early neurodevelopmental processes are only marginally affected. These findings underscore the potential of metal oxide NPs and DEPs for widespread interference with the placental secretome and identify vascular morphogenesis as a sensitive outcome for the indirect developmental toxicity of different NPs. Overall, this work has profound implications for the future safety assessment of NPs for industrial, commercial, or medical applications in pregnancy, which should consider placenta‐mediated toxicity by holistic secretomics approaches to ensure the development of safe nanotechnologies.

## Graphical abstract

Prenatal exposure of nanoparticles (SiO_2_, TiO_2_, diesel exhaust particles) at the human placenta induces material‐ and pregnancy stage‐specific dysregulation of placental signaling including secreted inflammatory, vascular, and endocrine mediators as well as extracellular vesicle‐associated proteins. The altered secretome produces pronounced anti‐angiogenic and anti‐vasculogenic effects, while early neurodevelopmental processes are only marginally affected.

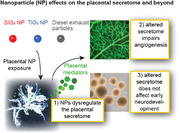

## 1 Introduction

Nanotechnological advances continue to foster the global production and widespread application of engineered nanoparticles (NPs) in many fields including nanomedicine, food, agriculture, textiles, cosmetics, energy, or electronics.^[^ ^1^ ^]^ The ever‐increasing exposure to NPs poses risks to human health, in particular for sensitive populations such as expecting mothers and their unborn children. In pregnancy, a tightly regulated crosstalk between maternal and fetal tissues and the interfacing placental tissue is imperative to ensure successful pregnancy outcomes. Here, the placenta takes center stage due to its numerous vital functions (e.g., nutrition/gas exchange or endocrine, immunologic, metabolic, and protective barrier functions). Unsurprisingly, abnormal placental development and functional impairments are therefore associated with numerous pregnancy complications such as preeclampsia (PE), intrauterine growth restriction (IUGR), stillbirth, placental abruption, and preterm birth (PTB).^[^ ^2^ ^]^

There is ample evidence that NPs can induce developmental toxicity,^[^ ^3^ ^]^ however, the involved mechanisms are largely unknown. The current paradigm often favors maternal‐fetal particle transfer and the direct effects of translocated particles to fetal tissues. However, NPs could also indirectly harm the developing fetus if they accumulate in the placenta and interfere with essential tissue functions and the release of signaling factors.^[^ ^4^ ^]^ This can be critical since an inappropriate or imbalanced secretion of placental hormones, cytokines, and angiogenic factors is involved in the pathogenesis of numerous pregnancy disorders such as PE, IUGR, or PTB.^[^ ^5^, ^6^, ^7^ ^]^ For instance, exposure to cobalt‐chromium NPs (40 µg mL^−1^) has been shown to induce indirect DNA damage to fibroblasts, which was mediated by oxidative stress responses and placental transmission of purine nucleotides (e.g., ATP) via connexin gap junctions.^[^ ^8^, ^9^ ^]^ In a follow‐up study, further insights into the developmental toxicity mechanisms were achieved revealing that placental CoCr NPs triggered impairment of the autophagic flux and the release of interleukin‐6 (IL‐6), which affected differentiation of human neural progenitor cells and DNA damage in the derived neurons and astrocytes.^[^ ^10^ ^]^ These seminal studies substantiate the potential of NPs to induce indirect placenta‐mediated embryo‐/fetotoxicity. However, further investigations are urgently warranted to unravel the full extent of NP interference with the entire placental secretome, to discern differences between early versus late placental signaling (gestational‐stage dependency) or between different NPs types (material‐dependency), and to identify additional developmentally relevant processes affected by an altered placental secretome. Angiogenesis, vasculogenesis, and neurodevelopment are among the key processes for proper placenta and embryo‐fetal development, which are orchestrated by factors secreted from the placenta.^[^ ^11^ ^]^ In fact, there is evidence from animal studies that TiO_2_ NPs can alter the uterine vascular anatomy and physiology^[^ ^12^ ^]^ or induce deficits in social behavior similar to autism spectrum disorder.^[^ ^13^ ^]^

Among the NPs of increasing concern for pregnancy exposure are large‐scale produced metal oxide NPs found in many consumer products and combustion‐derived air pollution particles. In an animal study, TiO_2_ and SiO_2_ NPs induced placental dysfunction, reduced fetal weight, or led to higher fetal resorption.^[^ ^14^ ^]^ Furthermore, epidemiological studies associated air pollution particles with IUGR,^[^ ^15^ ^]^ autism spectrum disorder^[^ ^16^ ^]^ as well as prenatal complications and postnatal diseases related to the respiratory system.^[^ ^17^ ^]^ Importantly, these particles have shown limited fetal transfer but accumulated in the placenta,^[^ ^14^, ^18^, ^19^ ^]^ pointing toward an indirect placenta‐mediated fetotoxicity effect. However, the developmental toxicity mechanisms of TiO_2_, SiO_2_ NPs, and DEPs and specifically, the contribution of placental mediators, are still largely unknown. In this study, we examined the effects of these environmentally or food‐relevant NPs on the human placental secretome and found interference with the secretion of multiple vascular, endocrine, and immune‐/inflammatory mediators in a material‐ and gestational stage‐specific manner. Dysregulation of placenta‐mediated signaling pathways was more pronounced in early placental tissue, a critical time window when embryo‐fetal development is strongly dependent on external cues for healthy development. To further understand if the combined changes in the placental secretome are sufficient to affect biological processes relevant to embryo‐fetal development, we examined the effects of conditioned placental media on angiogenesis and early neurodevelopment. While no effects were observed on neural progenitor cell proliferation or neuronal and astrocyte differentiation and migration, TiO_2_, SiO_2_ NP, and DEPs‐conditioned placental explant medium impaired angiogenesis and vascular network formation. Overall, these insights may lead to the identification of further critical nanoparticulate pollutants, the discovery of the origins of diseases primed from adverse early life exposures, the development of novel protection strategies or safe nanotherapies in pregnancy, and ultimately, a healthier next generation.

## 2 Results and Discussion

### 2.1 Deciphering Placenta‐Mediated Indirect Fetotoxicity Mechanisms

**Figure 1:**
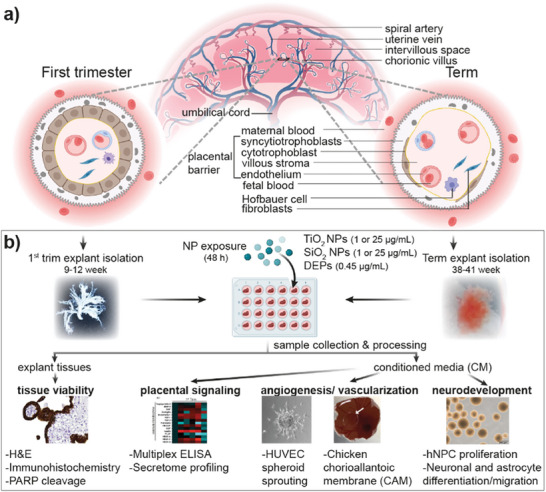
Study workflow. a) Schematic representation of the placental structure at first trimester and term. b) Experimental strategy showing the main steps of the study. H&E: hematoxylin‐eosin; hNPC: human neural progenitor cells; HUVEC: human umbilical cord endothelial cells; NP: nanoparticles; trim: trimester; PARP: poly(ADP‐ribosyl) polymerase.

The placenta is often considered the most species‐specific organ with a unique structure (**Figure** **1**a) and function. Therefore, we used placental explant cultures isolated from human first trimester and term placentae as a near‐physiological placental model to obtain human‐relevant results. Our approach to decipher how NPs induce potential placenta‐mediated indirect fetotoxicity involved the assessment of NP effects on placental viability, release of signaling factors and the subsequent effects of the altered placental secretome on angiogenesis/vascularization and early neurodevelopmental processes relevant to placental and embryo‐fetal development (Figure 1b). For this study, we selected TiO_2_, SiO_2_ NPs, and DEPs, where previous studies have found adverse pregnancy outcomes with only low or absent fetal particle transfer.^[^ ^14^, ^20^, ^21^ ^]^ We carefully characterized all particles and their characteristics are summarized in Figure S1a,b (Supporting Information). In an initial dose‐response study (NPs 5–100 µg mL^−1^; DEPs 0.45–10 µg mL^−1[^ ^21^ ^]^ due to assay interference at higher concentrations) in placental BeWo b30 trophoblast cells (Figure S1c, Supporting Information), we observed a slight reduction in cell viability only after exposure to high NP concentrations (50–100 µg mL^−1^). Since nano‐bio interactions are dose‐dependent, we aimed to study physiologically‐relevant concentrations to achieve human exposure‐relevant results. A recent study detected TiO_2_ and SiO_2_ NP in various human tissues with average concentration ranges of 0.01–1.8 mg k g^−1^ and <0.2–25 mg k g^−1^, respectively.^[^ ^22^ ^]^ For the human placenta, Ti concentrations of 0.01–0.48 mg k g^−1^ were reported,^[^ ^19^ ^]^ which is within the lower range of the previous study. Extrapolating to a 30 mg explant tissue, would correspond to 0.3–54 µg TiO_2_ NPs/explant or 6–750 µg SiO_2_ NPs/explant. We selected 1 and 25 µg mL^−1^ for TiO_2_ and SiO_2_ NPs (1.5 and 37.5 µg NPs/explant assuming 100% uptake) as physiologically realistic concentrations. For DEPs, a concentration of 0.45 µg mL^−1^ was selected based on extrapolation from previously determined values of 2.09 × 10^4^ ambient black carbon particles mm^−3^ in human placental tissue.^[^ ^21^, ^23^ ^]^ For the final studies on the downstream impact of the altered placental secretome on angiogenesis and early neurodevelopment, we depleted the remaining NPs from CM by high‐speed centrifugation (Figure S1d, Supporting Information). The removal of NPs from CM was confirmed by dynamic light scattering (DLS) measurement before and after centrifugation indicating the absence of NP‐associated peaks after the centrifugation step (Figure S1d, Supporting Information).

**Figure 2:**
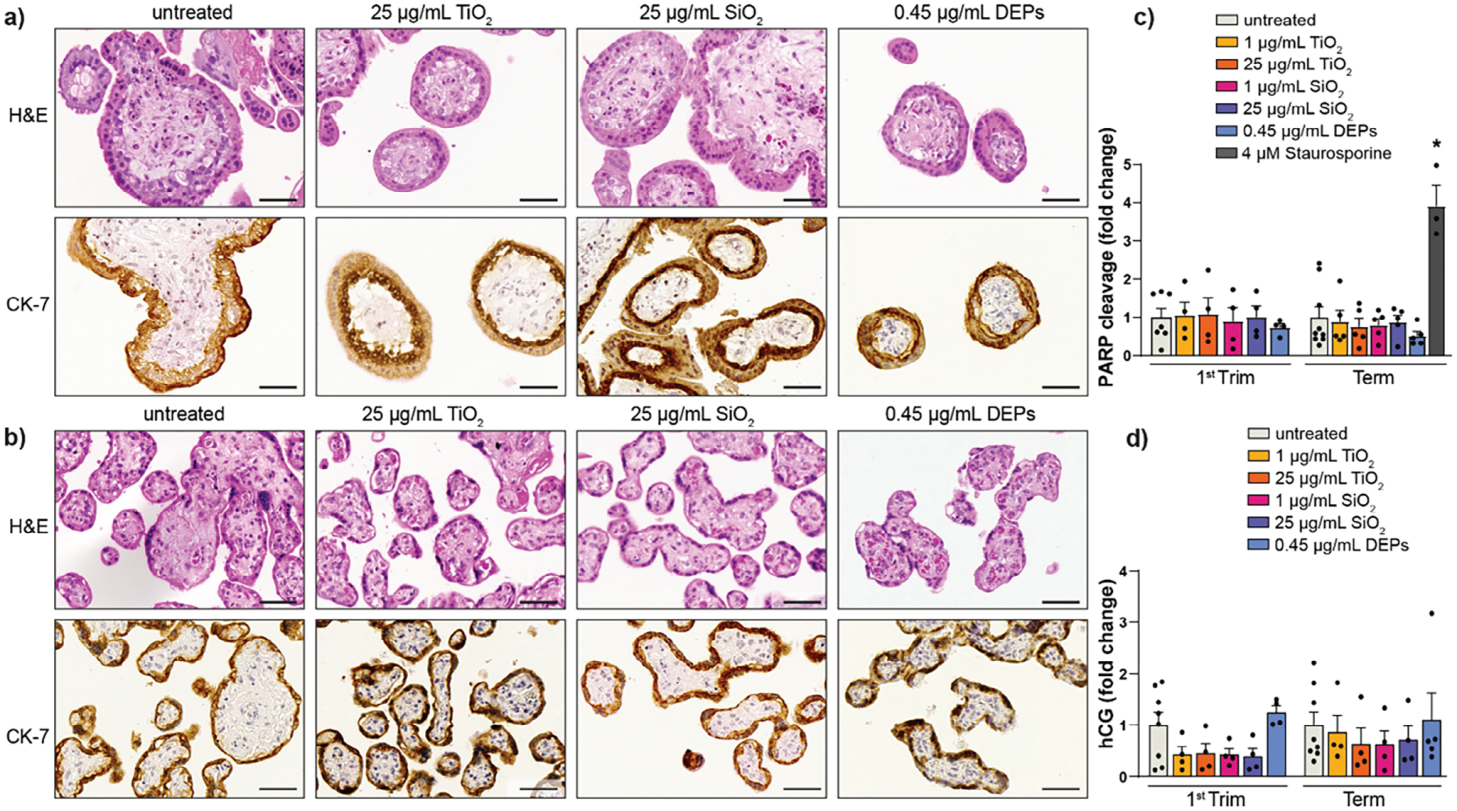
Effect of TiO_2_, SiO_2_ NPs, and DEPs on human placental tissue integrity, viability, and functionality after 48 h of exposure. Explants from first trimester (a) and term (b) placentae were exposed to TiO_2_, SiO_2_ NPs, (1 and 25 µg mL^−1^) or DEPs (0.45 µg mL^−1^) and characterized for histological and immunohistochemical changes by staining for H&E and trophoblast marker CK‐7, respectively. Representative images from three independent biological experiments are shown. Scale bars = 50 µm. c) PARP cleavage after 48 h of particle exposure in the tissue explants. Data represent the mean (± SEM) fold changes (FC) compared to the untreated control of n = 3–9 biologically independent samples (First trim: untreated n = 7, all others n = 4; Term: untreated n = 9, Staurosporine (positive control) n = 3, all others n = 5) d) Effect of NPs on hCG release from first trimester and term placental explants. Data represent the mean (± SEM) FC compared to the untreated control of n = 4–8 biologically independent samples (First trim: untreated n = 8, all others n = 4; Term: untreated n = 8, DEPs n = 5, all others n = 4). One‐way ANOVA with Dunnett's multiple comparisons correction was used for the analysis of comparisons between control (untreated) and the treatments (c) ^*^p < 0.0001. CK‐7: cytokeratin‐7; hCG: human chrionic gonadotropin; PARP: poly(ADP‐ribosyl) polymerase.

Before assessing the effects of NPs on placental signaling, we verified that placental explants maintained their tissue integrity and viability. Exposure of first trimester and term explants to TiO_2_, SiO_2_ NPs, or DEPs for 48 h did not induce any apparent damage to placental tissue integrity (**Figure** **2**a,b). Hematoxylin‐eosin (H&E) staining did not show any signs of pathological lesions and cytokeratin‐7 (CK‐7) staining revealed the presence of a continuous trophoblast bilayer (cytotrophoblast and overlying syncytiotrophoblast) in first trimester explants and a syncytiotrophoblast monolayer in term explants. The absence of NP‐induced cytotoxicity was further confirmed by a lack of increased poly(ADP‐ribose) polymerase (PARP) cleavage as a marker for apoptotic cell death (Figure 2c). We have previously shown that TiO_2_ NPs^[^ ^18^ ^]^ and DEPs^[^ ^21^ ^]^ can accumulate in the placenta, especially in trophoblast cells, and further observed that some NPs (CuO, CdTe, graphene‐based materials) can reduce the secretion of human chorionic gonadotropin (hCG) from the trophoblasts.^[^ ^24^, ^25^ ^]^ hCG is an essential pregnancy hormone that regulates placentation, angiogenesis, and fetal growth.^[^ ^26^ ^]^ A cohort‐based epidemiological study demonstrated that low hCG levels during the late first trimester are linked to reduced fetal growth and birth weight.^[^ ^27^ ^]^ Here, we observed a trend for a decrease in hCG secretion from first‐trimester explants exposed to TiO_2_ and SiO_2_ NPs, whereas no effects were observed in term explants or from DEP exposures (Figure 2d). While these results provided a first indication that NPs can induce pregnancy‐stage specific interference with endocrine placental signaling, we further embarked to investigate the impact of NPs on the global secretome of the placenta.

### 2.2 TiO_2_, SiO_2_ NPs, and DEPs Dysregulate the Placental Secretome

**Figure 3:**
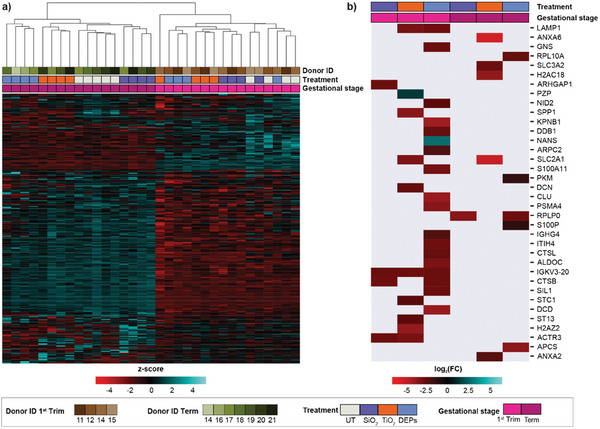
Impact of NPs on the placental secretome. a) Unsupervised hierarchical clustering of secreted proteins in CM of first trimester and term placental explants exposed to NPs for 48 h (n = 5 for term untreated; n = 4 for all other conditions). The rows and columns are clustered, while only the column dendrogram is displayed. Row intensities are z‐score scaled. b) Heatmap of significant (FDR < 0.1) differentially secreted proteins (absolute log_2_(FC) ≥ 1) in CM of first trimester and term placental explants exposed to NPs, compared to corresponding untreated samples (UT) (Welch's test with Benjamini–Hochberg multiple comparisons correction). Cells within the heatmap represent the log_2_(FC) values.

The placental secretome consists of a broad range of signaling and growth factors, hormones, and immune modulators that are important for determining pregnancy outcomes and potential pregnancy complications.^[^ ^28^ ^]^ To study the effects of TiO_2_, SiO_2_ NPs, and DEPs on the placental secretome during early and late pregnancy, we employed an unbiased secretome analysis to identify placental factors that could be involved in mediating indirect embryo‐fetotoxicity. First trimester and term explants were exposed to TiO_2_ and SiO_2_ NPs (25 µg mL^−1^) or DEPs (0.45 µg mL^−1^) for 48 h and the placental secretome was subjected to ultraperformance liquid chromatography‐tandem mass spectrometry (UPLC‐MS/MS) based label‐free quantification (LFQ) analysis in the data‐dependent acquisition (DDA) mode. First, we assessed whether the protein profiles reflected treatment stages and gestational stages (**Figure** **3**a). We found that unsupervised hierarchical clustering of secreted proteins clearly separated first trimester and term placental explants. Further subclustering for term samples correctly grouped individual treatments with different NPs. However, individual treatments on the first trimester samples partially overlapped in this analysis, thus suggesting that the interdonor variability also had a pronounced influence on the secretome. The higher variability in the first trimester samples could also be because the placenta undergoes rapid structural and functional changes in early pregnancy and thus small differences in the gestational age between donors (7–10 weeks) may result in different NP responses.

For each treatment, we identified the proteins with significant (false discovery rate (FDR) < 0.1) differences (absolute log2 fold changes (FC) ≥ 1) compared to untreated explants. A total of 1430 and 1398 proteins were detected in CM of first trimester and term explants, respectively. We found that 27 proteins in the first trimester (1.89% of all measured proteins) and ten proteins in term (0.72% of measured proteins) had different expression levels in CMs after the treatment with NPs (Figure 3b). In addition, CM of DEPs‐treated first trimester explants had the highest number of differentially expressed proteins (18 proteins) when compared to non‐treated CMs. Of note, only one protein (SLC2A1 or GLUT‐1) was found differentially regulated in CMs of both first trimester and term samples by TiO_2_ NP exposure. This further highlights the gestational stage‐dependent response to the selected NPs. Glucose transporter 1 (GLUT1) is expressed in the ST and facilitates the maternal‐fetal transport of glucose essential for normal fetal growth.^[^ ^29^ ^]^ Recently, placental expression of GLUT1 was found to be down‐regulated at the apical plasma membrane of the syncytiotrophoblast in PE but further studies are warranted to understand if this contributes to the development of IUGR in PE.^[^ ^30^ ^]^ Although GLUT1 is a transmembrane transporter, its detection in the medium could be due to the shedding of EVs containing GLUT1. Interestingly, GLUT1‐positive EVs were recently identified to be secreted from human endometrial stromal cells and could promote decidualization, angiogenesis and trophoblast differentiation in recipient cells.^[^ ^31^ ^]^ Similar paracrine signaling via EVs might also occur between the placenta and fetal tissues and our study provides first hints that such processes could be affected by in‐utero exposure to NPs. While the majority of proteins were secreted in a material‐specific manner, expression levels of the IGKV3‐20 (immunoglobulin kappa variable 3–20) protein were affected similarly by all three NP types in first trimester explants. IGKV3‐20 encodes the V region of the variable domain of immunoglobulin light chains that participate in antigen recognition. While information on pregnancy is scarce, it has been found that IGKV3‐20 was up‐regulated in placenta accreta spectrum (PAS) cases compared to control cases, indicating its relevance in discriminating between PAS and control groups.^[^ ^32^ ^]^

Several of the placental factors detected in this profiling study are known to play roles during pregnancy and/or have been previously associated with pregnancy complications, which suggests their importance for successful pregnancy outcomes. For example, osteopontin (SPP1), which plays a critical role during embryo implantation and placentation,^[^ ^33^ ^]^ was down‐regulated specifically by TiO_2_ NP exposure in first‐trimester explants. Moreover, secretion levels of lysosomal proteases such as cathepsin B (CTSB) or procathepsin L (CTSL) were decreased in early explants after exposure to DEPs and SiO_2_ NPs, respectively. A previous study conducted in pigs showed that the release of both CTSB and CTSL helps in remodeling the placenta.^[^ ^34^ ^]^ It is also interesting to note that others have found a crucial regulatory function of lysosomal cathepsins (i.e., CTSL1, CTSB) in immunomodulation^[^ ^35^ ^]^ and vascular remodeling of placental tissue.^[^ ^34^ ^]^ Therefore, we further performed targeted multiplex profiling of angiogenic and immunomodulatory mediators.

### 2.3 TiO_2_, SiO_2_ NPs, and DEPs Alter the Secretion of Placental Immune/Inflammatory Factors

**Figure 4:**
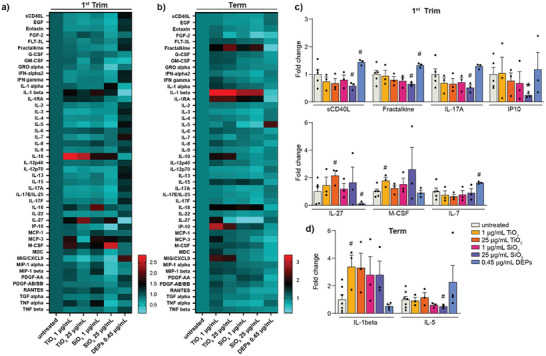
Impact of TiO_2_, SiO_2_ NPs, and DEPs on the secretion of cytokines and chemokines. Heatmaps of multiplex array results for 47 cytokine and chemokines from culture media of first trimester (a) and term human placental explants (b) after 48 h treatment with TiO_2_, SiO_2_ NPs, and DEPs. Color bars show values in FC relative to untreated controls. c,d) Release of selected angiogenic factors and cytokines/chemokines from first trimester (c) or term explants (d), which showed significant (^*^p < 0.05) or notable (#p < 0.09) changes compared to untreated controls (unpaired Student's *t*‐test, two‐sided); c) upper graph left to right: #p = 0.0837, 0.0705, 0.0695, 0.0852, 0.0749, ^*^p = 0.0350; lower graph left to right: #p = 0.0746, 0.0856, 0.0609, 0.0626; d) left to right: #p = 0.0879, 0.0813). Bar charts present mean (± SEM) from 3 to 7 independent experiments (First trim: untreated n = 5, all others n = 3; Term: untreated n = 7, DEPs n = 4, all others n = 3).

The homeostatic balance between pro‐ and anti‐inflammatory T‐cell subsets is of key importance to achieve successful pregnancy since they regulate vascularization and immune tolerance during pregnancy. Unintentional effects on T‐cell immunity have been shown to cause recurrent miscarriages and potentially link to preeclampsia.^[^ ^36^ ^]^ To understand whether TiO_2_, SiO_2_ NPs, or DEP exposure of placental explants affects inflammatory/immune signaling, we performed 48‐plex multiplex array studies to analyze released cytokines in an explant medium. As shown in **Figure** **4**, effects were predominantly observed in first‐trimester explants. We detected trends (p < 0.09) of increased secretion of IL‐27 and M‐CSF, while sCD40L, IL‐17A, fractalkine, and IP‐10 were reduced after exposure to TiO_2_ and SiO_2_ NPs (Figure 3a,c). In contrast, DEPs induced a notable (p < 0.09) increase in sCD40L, fractalkine, and IL‐7 and a decrease in IL‐27 concentrations (Figure 4c). While responses were more similar for TiO_2_ and SiO_2_ NPs, DEPs affected different cytokines/chemokines, which might be due to organic compounds or metals adsorbed to the elemental carbon core. In term explants, TiO_2_ and SiO_2_ NPs increased the secretion of IL‐1β (Figure 4b,d). IL‐17A is a pro‐inflammatory cytokine secreted by specialized Th17 cells. It is believed that IL‐17A‐secreting cells support placental angiogenesis and protect the materno–fetal interface against extracellular microbes during early pregnancy.^[^ ^37^ ^]^ In addition, it has also been demonstrated that IL‐17A secretion could stimulate survival, proliferation, and invasion of human trophoblast cells during the first trimester of pregnancy.^[^ ^38^ ^]^ Interestingly, it has been shown that IL‐27 regulates the expression of IL‐17A by inhibiting the Th17 differentiation antagonistically, thereby reducing IL‐17A secretion.^[^ ^39^ ^]^ Here, an increased secretion of IL‐27 (a trend observed, Figure 4c) after TiO_2_ and SiO_2_ NPs exposure may be responsible for lowering the secretion of IL‐17 as compared to untreated controls. In a previous study, IL‐6 secretion from placental BeWo trophoblast bilayer barriers exposed to CoCr NPs was found to be involved in indirect DNA damage signaling across the placental barrier,^[^ ^10^ ^]^ but other cytokines/chemokines were not affected.^[^ ^9^ ^]^ Here, we observed the deregulation of multiple chemokines/cytokines, possibly due to the selection of different NPs and/or the use of placental explant cultures where other cells could contribute to the response besides the trophoblasts. Further studies are needed to identify the involved cell types, e.g., using (imaging) mass cytometry approaches.^[^ ^40^, ^41^ ^]^

### 2.4 TiO_2_, SiO_2_ NPs, and DEPs Impair Vasculo‐ and Angiogenic Signaling in Early‐Stage Placenta

**Figure 5:**
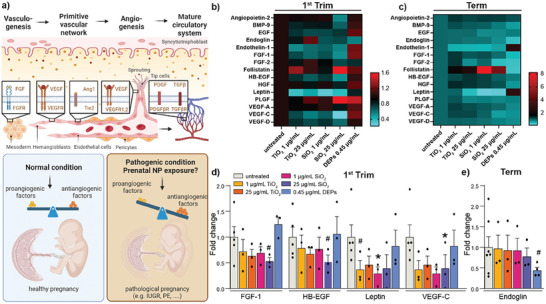
Impact of TiO_2_, SiO_2_ NPs, and DEPs on the secretion of vasculo‐ and angiogenic signaling factors. a) Scheme of the major paracrine vasculo‐and angiogenic factors involved in the formation of functional circulatory systems and hypothesis of NP‐induced adverse effect on maternal, placental, and embryo‐fetal vasculature from disruption of the angiogenic homeostasis. b,c) Heatmaps of multiplex array results for 15 angiogenic/hormone factors from culture media of first trimester (b) and term human placental explants (c) after 48 h treatment with TiO_2_, SiO_2_ NPs, and DEPs. Color bars show values in FC relative to untreated controls. Release of selected angiogenic/hormone factors from first trimester (d) or term explants (e), which showed significant (^*^p < 0.05) or notable changes (#p < 0.09) compared to untreated controls (unpaired Student's *t*‐test, two‐sided); d) left to right: #p = 0.0804, 0.0852, 0.0779, ^*^p = 0.0422, 0.0138; e) #p = 0.0752). Bar charts present mean (± SEM) from 3 to 7 independent experiments (First trim: untreated n = 5, all others n = 3; Term: untreated n = 7, DEPs n = 4, all others n = 3). IUGR: intrauterine growth restriction; PE: preeclampsia.

Angiogenesis and vascularization during early gestation are crucial for placentation, fetal development, and successful pregnancy. As a corollary, impaired vascularization has been considered a primary cause of pre‐eclampsia^[^ ^42^ ^]^ (**Figure** **5**a). These processes are tightly regulated by vasculogenic and angiogenic growth factors (e.g., VEGF A to D, FGF) secreted from placental cells, especially trophoblasts, during early gestation.^[^ ^43^ ^]^ Genetic and biochemical screenings of VEGF functions in mice revealed that an impaired expression or release of these factors causes placental pathology and abnormal embryo development.^[^ ^44^ ^]^ Here, we performed 17‐plex angiogenesis and growth factor array studies and observed trends (p < 0.09) or statistical significance (p < 0.05) in reduced secretion of several vasculo‐ and angiogenic factors (FGF‐1, VEGF‐C, HB‐EGF, leptin) after exposure to TiO_2_ and SiO_2_ NPs in first trimester explants (Figure 5b,d). HB‐EGF is an important growth factor with cytoprotective function, especially during early pregnancy, and its reduced expression is involved in pre‐eclampsia.^[^ ^45^ ^]^ Leptin also plays a crucial function during early pregnancy in placental signaling, immunomodulation, proliferation, and invasion. Dysregulation of leptin content during early placentation has been associated with polycystic ovary syndrome, recurrent miscarriage, gestational diabetes mellitus, PE, and IUGR.^[^ ^46^ ^]^ In term placental explants, we found that secretion of these factors was not affected after TiO_2_ and SiO_2_ NP exposure (Figure 5c). DEPs exposure exclusively dysregulated the release of endoglin in term placenta (Figure 5e). Overall, NP‐mediated dysregulation in the secretion of placental vasculo‐/angiogenic factors and hormones during early pregnancy may impair proper placental and embryo‐fetal development. It is important to acknowledge that placental factors do not affect only one pathway (e.g., inflammation, vascularization, or endocrine function) but that they can have pleiotropic effects and interlink with multiple pathways. For instance, IL‐1 family cytokines not only induce inflammatory responses but can also mediate angiogenesis, either directly (by binding and activating its receptor on endothelial cells) or indirectly via induction of proangiogenic factors such as VEGF.^[^ ^47^ ^]^ In pregnant rats, systemic inflammation (IL‐4 and IL‐6) was suggested to be involved in TiO_2_ NP‐induced maternal and fetal microvascular dysfunction.^[^ ^48^ ^]^ Another putative interconnection between endocrine and vascular signaling pathways has been proposed from an intravenous injection study of oxidized multiwalled carbon nanotubes (oMWCNTs) in pregnant mice, which observed deregulation of progesterone and estradiol levels along with decreased VEGF levels and placental vascularization.^[^ ^49^ ^]^

To achieve a better understanding of the indirect developmental toxicity resulting from the NP‐induced changes in the placental secretome, we further investigated the impact of the CM (concerted action of all deregulated proteins) on two key processes relevant to proper embryo‐fetal development and maternal–fetal health, namely angiogenesis and neurodevelopment.

### 2.5 Altered Placental Secretome Impairs Angiogenesis/Vascularization

**Figure 6:**
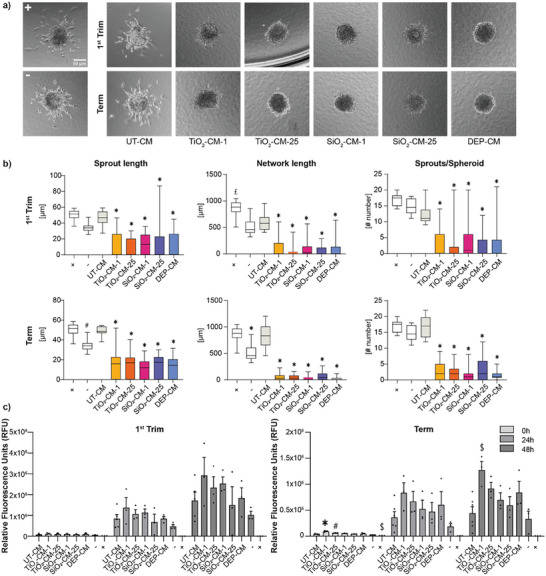
Effect of first trimester and term CM on HUVEC spheroid sprouting. a) Placental explant tissues from the first trimester or term were treated with 1 or 25 µg mL^−1^ TiO_2_ or SiO_2_ NPs or 0.45 µg mL^−1^ DEPs for 48 h and NP‐depleted CM was added to HUVEC spheroids to detect potential changes in spheroid sprouting during 48 h of cultivation. The sprouting assay was performed with CM from 4 to 5 independent NP explant exposures (TiO_2_ and SiO_2_ NPs n = 4; DEPs First trim n = 5; DEPs term n = 4; UT‐CM n = 1 pooled CM from 4 explants; +, – n = 1) and a total of 8–38 spheroids per condition were measured. Representative images are shown. (+) = positive control (20 ng mL^−1^ bFGF); (−) = negative control (medium only). b) Quantification of sprouting assays for sprout length, network length, and number of sprouts per spheroid. Data show mean (± SEM). #p = 0.128, £p = 0.0005, ^*^p < 0.0001 compared to UT‐CM (one‐way ANOVA with Dunnett's multiple comparisons correction). c) Viability of HUVECs treated with first trimester and term CM after 0, 24, and 48 h using RT‐Glo viability assay. (+) = positive control (1% Triton X‐100); (−) = negative control (TM with 20% EGM‐2). Data represent mean (± SEM) from at least three independent experiments for each treatment (First Trim: UT‐CM n = 5, Term: UT‐CM n = 6, all others n = 3). One‐way ANOVA with Dunnett's multiple comparisons correction was used for the analysis of comparisons between the control and the treatments (#p = 0.0125; from left to right $p = 0.0022, 0.0026; ^*^p < 0.0001). CM: conditioned medium; UT: untreated.

To understand potential effects on angiogenesis, CM was applied to human umbilical vein endothelial cells (HUVEC) spheroids embedded in a 3D collagen environment for 48 h and spheroid sprouting was analyzed (**Figure** **6**). 3D models of vascular morphogenesis including HUVEC spheroid sprouting are frequently used as high‐throughput in vitro screening model to detect interference with endothelial cell differentiation, migration, proliferation, aggregation, and rearrangement of these cells to form cords.^[^ ^50^, ^51^ ^]^ Exposure to endothelial growth medium‐2 (EGM‐2, negative control) resulted in an average sprout length of 35 µm, whereas basic fibroblast growth factor (bFGF, positive control) increased the mean sprout length to 51 µm (Figure 6a,b). In the presence of CM from untreated first trimester or term explants (UT‐CM), spheroids displayed a slightly higher average sprout length of 46 or 50 µm, respectively, which is most likely due to the presence of pro‐angiogenic placental signaling factors in the CM. Sprout length was significantly reduced in the presence of CM from NP‐treated explants (NP‐CM) compared to CM from untreated controls for both, the first trimester and term explants. The observed NP‐CM‐induced anti‐sprouting effects were also evident from a significant decrease in sprout length and total network length and a decline in the number of sprouts per spheroid (Figure 6b). The anti‐angiogenic effect was not due to cytotoxicity from the NP‐CM since the viability of HUVEC cells was not reduced compared to UT‐CM (Figure 6c).

**Figure 7:**
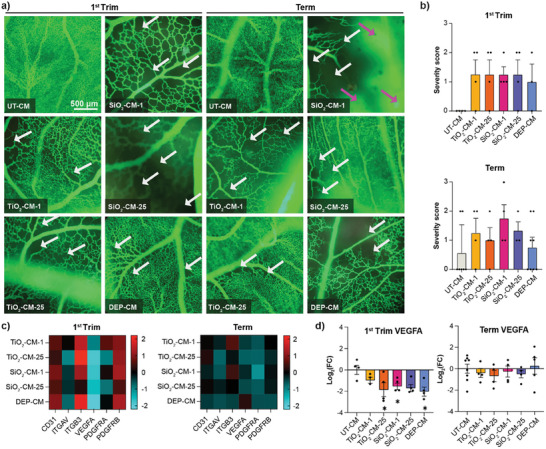
Impact of CM from TiO_2_, SiO_2_ NPs or DEPs exposed first trimester and term placental explants on CAM vasculature. Placental explant tissues were treated with NP‐free tissue medium, 1 or 25 µg mL^−1^ TiO_2_, SiO_2_ NPs, or 0.45 µg mL^−1^ DEPs for 48 h and CM (referred to as UT‐CM, TiO_2_‐CM‐1, TiO_2_‐CM‐25, SiO_2_‐CM‐1, SiO_2_‐CM‐25, DEP‐CM) were added onto CAM for 48 h (2 × 24 h) before injection of green fluorescent dextran and microscopic analysis to visualize vascular networks. a) One selected image per treatment representing the most striking effect observed on vascular network formation including enlarged avascular zones (represented by black zones; white arrows) with decreased vessel density and vascular leakage (magenta arrows). b) Scoring of vascular effects (0 = no effects; 1 = mild: moderately decreased vessel density; 2 = moderate: strongly decreased vessel density; 3 = severe: strongly decreased vessel density & vascular leakage). Data show mean (± SEM) from at least three independent experiments per condition (First trim: DEP‐CM n = 3, all others n = 4; Term: UT‐CM n = 7, TiO_2_‐CM‐1 and TiO_2_‐CM‐25 n = 4, SiO_2_‐CM‐1 and SiO_2_‐CM‐25 n = 3, DEP‐CM n = 5). No significant differences (one‐way ANOVA with Dunnett's multiple comparisons correction). c,d) CAM mRNA expression profile after treatment with first trimester or term CM for a selection of genes involved in angiogenesis by qPCR. Gene expression was related to reference genes, and subsequently normalized to untreated CAMs. c) Heatmaps show log_2_(FC) values relative to UT‐CM exposed CAM, with up‐regulation of gene expression in red, and down‐regulation in blue. Colors represent average log_2_(FC) values. d) Graphs highlight VEGFA expression in individual samples relative to UT‐CM‐exposed CAM. b,d) Data show mean (± SEM) from at least three independent experiments per condition (First trim: TiO_2_‐CM‐1 n = 3, all others n = 4; Term: UT‐CM n = 8, DEP‐CM n = 5, all others n = 4). Left to right:*p = 0.0276, 0.0437, 0.0146 compared to UT‐CM (one‐way ANOVA with Dunnett's multiple comparisons correction). CM: conditioned medium; UT: untreated.

To verify the anti‐angiogenic effects, as well as the potential impact on vasculogenesis, the chicken chorioallantoic membrane (CAM), was used to investigate vascular development and morphology as a response to CM from NP‐exposed placental explants. The CAM is a highly vascularized extra‐embryonic membrane that offers an advantageous platform to investigate vascular development and morphology in response to experimental substances but also hemodynamics, immune cell trafficking, transplantation, and therapeutic responses. It is a cost‐effective preclinical in vivo model with key advantages related to easy accessibility, optical transparency and rapid growth of its vascular network.^[^ ^52^ ^]^ At day 7 post‐fertilization, a 5 mm ring was placed onto the vascularized membrane to confine the treatment and CM was added twice (0 and 24 h) before analyzing the effects on vascularization at 48 h. CAMs treated with CM from untreated control explants displayed a dense network of blood vessels (**Figure** **7**a). In contrast, exposure to the CM from NP‐exposed first trimester and term placental explants showed evidence of adverse effects on vasculogenesis. This was apparent from enlarged avascular zones (black areas on the fluorescent angiograms) between individual vessels indicating a mild to severe decrease in vessel density, branching points, and in some cases a clear reduction in vessel lengths. The most severe effect was observed from treatment with the term SiO_2_‐CM (1 µg mL^−1^; SiO_2_‐CM‐1), which additionally led to vessel leakage in CAM. However, a severity scoring of all individual experiments revealed a high donor variability resulting in an overall trend but no significance of the observed vascular effects (Figure 7b). The variability could not only have resulted from differences between placenta donors but also from inherent variabilities in vascularization in the CAM from different eggs. Following treatment of CAM with the CM of NP‐treated placental tissues, CAM sections were excised and processed for qPCR profiling of selected genes (CD31, ITGAV, ITGB3, VEGFA, PDFGRA, PDFGRB) involved in developmental angiogenesis. First‐trimester NP‐CM had a more pronounced impact on the expression of angiogenic genes in CAM than the term NP‐CM (Figure 7c,d). Notably, secreted factors in the first trimester NP‐CM caused a significant down‐regulation of the prime pro‐angiogenic factor VEGF‐A. The significant decrease in VEGF‐A implies an impaired angiogenic response after exposure to NP‐CM and concomitant reduction of vascularization. Intriguingly, the expression of several other genes was not changed accordingly, which may indicate the onset of compensatory mechanisms to rescue effects induced by NP‐CM. Furthermore, effects were less pronounced in CAMs exposed to term NP‐CMs, suggesting that TiO_2_, SiO_2_ NPs, and DEPs have divergent effects depending on the time of exposure during pregnancy.

### 2.6 Altered Placental Secretome has Negligible Effects on Early Neurodevelopment

**Figure 8:**
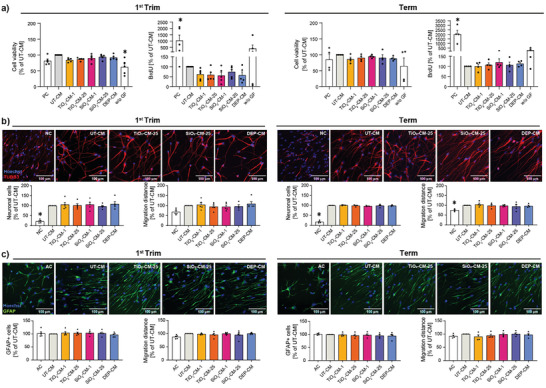
Impact of CM from TiO_2_, SiO_2_ NPs or DEPs exposed first trimester and term placental explants on neurodevelopmental processes. a) hNPC viability and proliferation in the presence of CM from First trimester or term explants. b) Impact of CM on neuronal differentiation assessed from immunofluorescent staining of TUBB3+ neurons and quantified as percentage of TUBB3+ neurons and neuronal migration distance. c) Impact of CM on astrocyte differentiation assessed from immunofluorescent staining of GFAP+ astrocytes and quantified as percentage of GFAP+ astrocytes and astrocyte migration distance. Representative fluorescent images are shown. CM samples from individual placenta donors were defined as independent biological replicates. Data show mean (± SEM) from at least four independent experiments per condition (First trim: neuronal migration distance SiO_2_‐CM‐25 n = 4, all others n = 5, term: n = 4). ^*^p < 0.05 (a) from left to right: p < 0.0001, p = 0.0050, p < 0.0001; b) from left to right: p < 0.0001, p < 0.0001, p = 0.0271) compared to UT‐CM (one‐way ANOVA with Dunnett's multiple comparisons correction). CM: conditioned medium; UT: untreated; PC: proliferation control; NC: neuron differentiation control; AC: astrocyte differentiation control, w/o GF: without growth factor control.

During early brain development, proliferation represents one pivotal cellular process ensuring that an adequate number of cells are generated to form functional neural networks.^[^ ^53^ ^]^ We first confirmed that CM (UT‐CM or NP‐CM) did not affect the viability of proliferating human neural progenitor cells (hNPCs) as compared to cells cultivated in a proliferation medium (**Figure** **8**a). In the presence of CM, we observed a considerably reduced hNPC proliferation already from UT‐CM. NP‐CM from first trimester but not term explants induced a further slight decrease in the proliferation rate indicating a modest gestational stage‐dependent effect. During neurodevelopment, proliferation is regulated by various signaling pathways including EGFR and FGFR signaling.^[^ ^54^ ^]^ The reduced expression of HB‐EGF and FGF‐1 in the CM of NP‐treated placentae from the first trimester (Figure 5d) might be a potential cause for the slightly lower proliferation rate of hNPCs after cultivation in the respective NP‐CM compared to the UT‐CM. Other key events of functional CNS development are neuronal lineage fate commitment and neurite outgrowth, which are crucial for neuronal network formation and associated learning and memory functions.^[^ ^55^ ^]^ Here, we did not observe any significant differences between the NP‐CM and UT‐CM on the percentage of TUBB3+ neurons and their migration distance, indicating that the neurogenesis‐promoting factors secreted by the placenta were not affected by NP exposure (Figure 8b). We next assessed the impact of the CM on hNPC‐derived astrocytes by differentiating hNPCs for 5 days in the presence of BMP2 and CNTF. Astrocytes contribute significantly to the intricate orchestration of neural circuits and the formation of a functional CNS by regulating synapse formation, elimination, and function.^[^ ^56^ ^]^ We did not observe significant effects on GFAP+ astrocyte numbers when astrocyte differentiation was performed in the presence of UT‐CM, NP‐CM, or DEPs‐CM collected from first trimester and term explants (Figure 8c). When assessing astrocyte inflammatory activation (immunocytochemical staining for ICAM‐1), we did not observe distinct differences in the expression of ICAM‐1 between the NP‐CM and the UT‐CM suggesting that the NP‐induced changes in placental secretome did not promote astrocyte activation (Figure S2, Supporting Information).

## 3 Conclusion

In this study, we uncovered that TiO_2_, SiO_2_ NPs and DEPs can induce widespread gestational stage‐specific perturbations of the placental secretome including dysregulation of multiple pro‐angio‐ and vasculogenic factors, hormones, and immunomodulatory cytokines/chemokines. Importantly, the selected NPs are of high relevance and potential concern for pregnancy exposure, and the effects were observed at physiologically realistic concentrations already from a single short‐term exposure. We showed that TiO_2_, SiO_2_ NP, and DEPs interference with placental signaling is complex and affects a multitude of secreted factors. Furthermore, we found that effects on placental signaling are material‐specific, with metal oxide NPs (TiO_2_ and SiO_2_ NPs) showing more similar responses to the placental secretome compared to DEPs. In addition, TiO_2_, SiO_2_ NP effects were more pronounced in early placental tissue (e.g., trend for a decrease of β‐hCG release). Although the differences were often relatively small, the combined alterations of the placental secretome may act synergistically to form a hostile environment for embryo‐fetal development. Indeed, we identified that NP‐induced alterations of the placental secretome had a major impact on angiogenesis and vascular network formation, which are indispensable processes for proper placental and embryo‐fetal development. While our study has some limitations such as the lack of an entire organism or dynamic exposure conditions, the use of ex vivo human placenta tissue from early and late‐stage pregnancy provides one of the most physiological experimental models to achieve human‐relevant results and avoids the considerable species‐specific differences in placental structure, function and physiology.^[^ ^57^, ^58^, ^59^ ^]^ Therefore our study provides interesting candidates for further verification in more complex models and future work is warranted to decipher the interaction networks of secreted placental factors involved in adverse pregnancy outcomes. Overall, our findings highlight the central contribution of the placenta and its secretome in the developmental toxicity of different types of NPs including metal‐oxide TiO_2_, SiO_2_ NPs, and DEPs containing organic and inorganic elements. Regarding the emerging field of gestational nanotherapies,^[^ ^60^, ^61^, ^62^, ^63^ ^]^ it will be particularly important to exclude potential indirect placenta‐mediated toxicity of medically relevant NPs (e.g., lipid and polymeric NPs), even if these NPs are generally considered more biocompatible than inorganic NPs. In fact, there is some evidence that previously perceived low‐toxicity polymeric NPs such as polystyrene NPs can dysregulate the placental transcriptome including expression of genes related to inflammation, iron homeostasis and xenobiotics detoxification (cytochrom P450),^[^ ^64^ ^]^ thus substantiating the need for a thorough hazard assessment of gestational nanomedicines including effects on the placental gene and protein expression level. We believe that our work will motivate a burst of novel research activities to unravel placenta‐mediated developmental toxicity mechanisms of NPs, which is imperative for the sustainable and safe use of nanotechnologies and the development of safe nanomedicines in pregnancy.

## 4 Experimental Section

### Chemicals and Reagents

All chemicals and reagents used in this study were obtained from Sigma–Aldrich unless stated otherwise.

### NP Dispersion

NP dispersions were prepared in tissue medium (TM), which is Dulbecco's modified eagle medium (DMEM) diluted with Earl's buffer in a 1:2 ratio, supplemented with bovine (BSA (10 g L^−1^), dextran 40 (10 g L^−1^), sodium heparin (17.5 mg L^−1^), and amoxicillin (250 mg L^−1^). DEPs were purchased from the National Institute for Standards and Technology (NIST, SRM1650b) and a stock dispersion of 45 µg mL^−1^ in TM was prepared using a probe sonicator operating at 230 V/50 Hz (Branson Sonifier 250, Branson Ultrasonic Co., probe diameter of 6.5 mm, maximum peak‐to‐peak amplitude of 247 µm) as previously described.^[^ ^21^ ^]^ Nanosized TiO_2_ powder (15 nm) was supplied by Nanostructured & Amorphous Materials, Inc. (5430MR, USA) and nanosized SiO_2_ (70 nm) was purchased from micromod Partikeltechnologie GmbH (43‐00‐701, Germany) as a suspension of 25 mg mL^−1^. Stock suspensions of 1 mg mL^−1^ in ultrapure water (MilliQ, >18 MΩ cm) were prepared freshly for each experiment using bath sonication (5 min, Sonorex RK156, Bandelin) and vortexed (1 min). For the secretomics study, dispersions were prepared in TM without BSA, since the high BSA content prevented the sensitive detection of secreted proteins. However, since the biocorona affects particle properties, biodistribution, uptake, and biological effects, NPs were pre‐coated with BSA by incubating stock suspension in complete TM with BSA at 37 °C for 1 h. NPs were centrifuged (25 000 g, 10 min), resuspended in MilliQ water by bath sonication (5 min), diluted to experimental concentrations in BSA‐free TM, and directly applied to the explants. Since DEPs dispersions could directly be prepared in TM and exhibited good stability over prolonged periods (up to 8 weeks), stock dispersions were directly diluted in TM without BSA resulting in a 100‐fold reduction in the BSA content. For neurodevelopmental studies, basal medium (BM; DMEM (#31966‐021, Thermo Fisher) and Ham's F12 (#31765‐027, Thermo Fisher) in a 2:1 ratio (v:v)) were used instead of TM to produce the NP dispersions. Methods for NP characterization are described in Supporting Information.

### Exposure of Human Placental Explants to NPs

First trimester (n = 16 gestational age 7–10 weeks) and term placentae (n = 15) were obtained from elective terminations of pregnancies or uncomplicated pregnancies after cesarean section from the Cantonal Hospital St. Gallen with written informed consent from the expecting mothers. The local ethics committee approved the study (EKOS 10/078; PB‐2018‐00069), which was performed according to the principles of the Declaration of Helsinki. Information on the sex was only obtained for the term placenta (female n = 7, male n = 8). Since access to the first trimester placenta was limited, the design and collection of sufficient donors to study sex‐specific responses were not pursued in this study. The tissue was cut into pieces of ≈3 mm in size and ≈30 mg in weight, placed in 24‐well plates (one explant per well), and cultivated in 1.5 mL freshly prepared NP working concentrations for 48 h under hypoxic conditions (5% CO_2_ and 8% O_2_) at 37 °C. Media (100 µL) was collected after 0, 6, 24, and 48 h of exposure and centrifuged (800 g, 10 min) to remove residual blood cells. Supernatants were transferred into new microtubes, centrifuged (25′000 g, 4 °C, 20 min) to remove NPs, and stored at −20 °C until further use (hCG ELISA and multiplexed profiling). After each sampling, wells were refilled with the same volumes of respective NP working concentrations. At the end of the experiment (48 h), tissues were snap‐frozen and stored at –80 °C until further processing (PARP cleavage ELISA). For (immuno)histological analysis, tissues were fixed in 8 mL of Roti‐Histofix 4% (Roth, P087.5) for 72 h at room temperature (RT). Potential interference from adsorption and co‐precipitation of placental mediators with the NPs as well as general NP interference responses with the biological assays were excluded (Supplementary information and Figure S3, Supporting Information).

### hCG ELISA

hCG ELISA was performed with CM collected from NP‐exposed placental explants or untreated controls as described previously.^[^ ^65^ ^]^ ELISA plates (96‐well; high binding, Corning, 9018) were pre‐coated with capture antibody rabbit anti‐ hCG (Dako, A0231; 1:10 000 in a 50mm NaHCO_3_ buffer) at 4 °C overnight. Plates were washed with 0.1%Tween‐20/PBS before applying blocking buffer (1% BSA/PBS) for 1.5 h at RT. hCGstandard (BioSupply UK, HOR‐250) was prepared as serial dilution from stock solution (1 µg mL^−1^ in 1% BSA/PBS) with 6000 pg mL^−1^ as the highest concentration, followed by 4000, 2000, 1000, 500, 250, 125, and 0 pg mL^−1^. After washing the plates with 0.1% Tween‐20/PBS, 100 µL of hCG standard and samples (CMs) from placental explant tissue cultures were pipetted into wells and incubated at 37 °C for 1 h in a humidified chamber. The plates were washed with 0.1% Tween‐ 20/PBS and exposed with 100 µL per well of detection antibody mouse anti‐hCG (Serotec,MCA 1436; 1:5000 in 1%BSA/PBS) for 1 h at 37 °C in a humidified chamber. After washing (0.1% Tween‐20/PBS), secondary detection antibody goat anti‐mouse IgG HRP (Biorad, 1706516; 1:5000 in 1% BSA/PBS) was added for 1 h at 37 °C in a humidified chamber. After washing (0.1% Tween‐20/PBS), 100 µL of TMP (3,3′,5,5′‐Tetramethylbenzidine) were applied per well and plates were incubated in the dark for 10 min at RT. Optical density (OD) was measured at 370 nm (BertholdTech Mithras2). The raw OD signals were normalized to whole tissue protein content, and the FC was calculated with reference to untreated CM. The experiments were run in technical duplicates.

### Cell Death

Cell death was determined by quantification of PARP cleavage in whole tissue lysates, prepared from first trimester and term explants, using PARP (Cleaved) [214/215] human ELISA Kit (KHO0741, Thermofisher) and following manufacturer's instructions. In brief, 50 µL of standards of known cleaved PARP concentrations and the tissue lysates were applied onto wells of pre‐coated 96‐well plate (monoclonal capture antibody specific for PARP (Cleaved) [214/215]). Cleaved PARP (50 µL) [214/215] detection antibody solution was added to each standard and sample and incubated for 3 h. After the washing step with the provided wash buffer, a secondary (rabbit) antibody was added for 30 min followed by incubation with the enzyme solution (horseradish peroxidase‐labeled anti‐rabbit IgG) for 30 min. After a final washing, substrate solution (TMB) was added for 30 min, and the reaction was terminated with a stop solution. Absorbance was measured on a plate reader at 450 nm (Mithras2 LB 943, Berthold Technologies GmbH). The optical signals were normalized to whole tissue protein content, and the FCs were calculated with reference to untreated CM. The experiments were run in technical duplicates.

### Histological and Immunohistochemical Characterization

Fixed placental tissue explants were embedded in paraffin and cut into 4 µm sections. Sections were deparaffinized and rehydrated by immersing them 3x for 10 min in Ottix Plus (Diapath, X0076) and 3x for 5 min in Ottix shaper (Diapath, X0096). For CK‐7 staining, heat‐induced antigen retrieval was performed at 95 °C for 15 min in 10 mm trisodium citrate buffer. To block peroxidases, slides were incubated in 3% hydrogen peroxide (H_2_O_2_) at RT for 15 min. After washing (2×5 min in PBS/0.05% Tween 20), blocking with 10% goat serum in antibody diluent (Dako AB diluent, S302281) was performed for 60 min at RT in a wet chamber. To block signals from endogenous avidin, biotin, and biotin‐binding proteins, Avidin–Biotin Block (Kit from VectorLabs, SP‐2001) was used as described by the manufacturer. Samples were incubated overnight with mouse anti‐CK7 (Dako; OV‐TL 12/30, 1:50 in Dako AB diluent). HRP‐conjugated goat‐anti‐mouse IgG (Vector Labs; #BA‐9200; 1:100 in PBS) was incubated with samples for 30 min, followed by immersion in ABC ELITE reagent (Vector Labs, Vectastain Elite ABC‐peroxidase Kit–standard, PK‐6100) for 30 min and DAB substrate (Vector Labs, DAB peroxidase substrate Kit, SK‐4100) for 1 min. Hematoxylin was used for counterstaining. Hematoxylin and eosin (H&E) staining was performed by immersing the de‐paraffinized sections in Hematoxylin Mayer for 10 min and then rinsing with running tap water for 10 min. After a 30 s wash with 1% acetic acid alcohol, the sections were immersed in Eosin for 30 s, rinsed with ultrapure water (MilliQ), rehydrated, and mounted with coverslips. Sections from a total of eight donors from the first trimester and term placenta samples were imaged.

### Multiplexed Profiling

The Discovery Assays Human Cytokine Array/Chemokine Array 48‐Plex and Human Angiogenesis Array & Growth Factor Array 17‐plex (Eve Technologies Corp, Calgary, Canada) were used to quantify 47 cytokine and chemokine biomarkers and 15 angiogenic biomarkers, respectively. The 47‐plex consisted of the following analytes: sCD40L, EGF, Eotaxin, FGF‐2, Flt‐3 ligand, Fractalkine, G‐CSF, GM‐CSF, GRO‐α, IFN α 2, IFNγ IL‐1α, IL‐1β, IL‐1ra, IL‐2, IL‐3, IL‐4, IL‐5, IL‐6, IL‐7, IL‐8, IL‐9, IL‐10, IL‐12p40, IL‐12p70, IL‐13, IL‐15, IL‐17A, IL‐17E/IL‐25, IL‐17F, IL‐18, IL‐22, IL‐27, IP‐10, MCP‐1, MCP‐3, M‐CSF, MDC (CCL22), MIG, MIP‐1α, MIP‐1β, PDGF‐AA, PDGF‐AB/BB, RANTES, TGFα, TNFα, and TNFβ. The 15‐plex consisted of the following analytes: Angiopoietin‐2, BMP‐9, EGF, Eng, Endothelin‐1, FGF‐1, FGF‐2, Follistatin, HB‐EGF, HGF, Leptin, PLGF, VEGF‐A, VEGF‐C, and VEGF‐D. CM was collected, centrifuged (25 000 × *g*, 4 °C, 20 min) to remove NPs and stored at −20 °C until further use. Analysis was done in duplicates without further dilution of the samples. The raw fluorescence intensity signals were normalized to whole tissue protein content and the FC were calculated with reference to untreated CM.

### Secretomics Measurements

CM of all treatments were collected and measured in two batches (first trimester and term placental explants). Protein concentrations of the samples were determined using the Lunatic UV/Vis polychromatic spectrophotometer (Unchained Labs). Protein (50 µg) from each sample were taken, reduced with 2 mm tris(2‐carboxyethyl)phosphine (TCEP) and alkylated with 15 mm chloroacetamide at 30 °C for 30 min. Subsequently, samples were processed using the single‐pot solid‐phase enhanced sample preparation (SP3).^[^ ^66^ ^]^ Protein purification, digestion as well as peptide clean‐up were performed using a KingFisher Flex System (Thermo Fisher Scientific) and Carboxylate‐Modified Magnetic Particles (GE Life Sciences; GE65152105050250, GE45152105050250). Following the manufacturer's instructions, beads were washed 3× with water at a concentration of 1 µg µL^−1^. After bead conditioning, samples were diluted with 100% ethanol to a final concentration of 50% ethanol. Beads, wash solutions, and the samples were loaded into 96 deep well‐ or microplates before transferring them to the KingFisher Flex System, where the following steps were carried out: collection of beads from the last washing step, protein binding to beads, 3× beads washing in 80% ethanol, protein digestion overnight at 37 °C with trypsin:protein ratio of 1:100 in 50 mm triethylammoniumbicarbonat (TEAB) and peptide elution from the magnetic beads using MilliQ water. Combined digest solution and water elution were dried to completeness and re‐solubilized in 20 µL MS sample buffer consisting of 3% acetonitrile and 0.1% formic acid. MS analysis was conducted on an Orbitrap Fusion (Thermo Scientific), coupled to a Digital PicoView source (New Objective) and an M‐Class UPLC (Waters). Channel A composed of 0.1% formic acid and channel B of 0.1% formic acid, 99.9% acetonitrile. UPLC‐MS/MS measurements were performed in randomized order for each sample individually, whereby 1 µL of peptides were used. Samples were loaded on a commercial MZ Symmetry C18 Trap Column (100 Å, 5 µm, 180 × 20 mm, Waters), which was followed by a nanoEase MZ C18 HSS T3 Column (100 Å, 1.8 µm, 75 × 250 mm, Waters). During chromatography, peptides were eluted with a constant flow rate of 300 nL min^−1^. The initial gradient of 5% B, held for 3 min, was increased within 80 min to a total of 24% B. In the next 10 min, B was further increased to 36%, followed by a 5 min washing step with 95% B. The column was finally re‐equilibrated to starting conditions for an additional 10 min. While measuring in DDA mode, the maximum cycle time was set to 3 s, the spray voltage to 2.3 kV, the funnel RF level to 60%, and capillary temperature to 275 °C. After accumulation to a target value of 400′000 or a maximum injection time of 50 ms, full scan MS spectra from 300 m/z to 1500 m/z were acquired at a resolution of 120′000 at 200 m/z. MS/MS spectra were recorded in the linear ion trap using quadrupole isolation with a window of 1.6 Da and higher energy C‐trap dissociation (HCD) fragmentation with 30% fragmentation energy while operating in rapid scan mode with a target value of 8′000 and a maximum injection time of 80 ms. Precursors exceeding the intensity of 5′000 were selected for MS/MS. In the settings, charge state screening was enabled. Single, unassigned, and charge states higher than seven were rejected. Previously for MS/MS selected precursor masses were excluded from further selection for 25 s, and the exclusion window was set at 10 ppm. Internal lock mass calibration on 371.1012 and 445.1200 m/z was used to acquire the samples.

### Secretomics Data Analysis

MS data was processed with the software MaxQuant (version 2.0.1.0),^[^ ^67^ ^]^ making use of the integrated Andromeda search engine for the subsequent protein identification. In doing so, the MS data were searched against a reference database compiled from Homo sapiens proteome sequences (UniProt (https://www.uniprot.org/) taxonomy 9606, canonical version from 2019‐07‐09), concatenated to its reversed decoyed FASTA database and common protein contaminants. While carbamidomethylation of cysteine amino acids was set as fixed, methionine oxidation and *N*‐terminal protein acetylation were set as variable protein modification. A minimum protein length of seven amino acids and a maximum of two missed‐cleavages were accepted. The enzyme specificity was set to trypsin/P according to the upstream protein digestion process. Default MaxQuant^[^ ^67^ ^]^ Orbitrap search settings were used. The maximum FDR was set to 0.01 for peptides and 0.05 for proteins. In addition, LFQ was enabled with a 2 min window between runs for matching. To obtain individual quantitative values, each file was kept separately. All MS spectra and relevant MaxQuant^[^ ^67^ ^]^ output tables have been deposited to the ProteomeXchange Consortium via the PRIDE (https://www.ebi.ac.uk/pride) partner repository with the dataset identifier PXD047037. When analyzing data of obtained secretomes from term placental explants, it was decided to exclude the untreated measurements of the donor IDs 14 and 16 due to the high number of unmeasured entries (over 95%). Corresponding information for the analysis was retrieved from the data reported in the proteinGroups.txt files. The analysis was conducted in Python (version 3.8.8). Commonly occurring contaminants, entries matching the reversed part of the decoy database, and proteins identified only by modification sites were excluded from the subsequent analysis. Furthermore, proteins were required to have been measured in at least 50% of the biological replicates in at least one of the conditions. Protein LFQ intensities were log2 transformed. After merging the two batches into a single DataFrame, through the “pandas' python library, proteins were restricted to secreted ones (Gene Ontology Cellular Compartment (GOCC): ‘secretory granule,” “secretory granule lumen,” “secretory granule membrane,” “extracellular vesicle,” “extracellular space,” “extracellular exosome”). In the next step, protein abundances were median centered to remove unwanted variations and discrete batch effects. Missing values were imputed separately for each sample following PhosR^[^ ^68^ ^]^ concepts, where missing values that were consistently absent in a condition and values missing only in a small fraction of samples in a condition were distinguished. Two normal distributions shifted left from the mean of the measured values were constructed. In cases where ≥50% of the replicates of a specific condition were measured, a distribution with a negative shift of 0.5 SD (standard deviation) of the original mean was constructed. Elsewhere, a distribution with a negative shift of 1.8 SD was constructed. In both cases, a width of 0.3 SD was selected. Missing data entries were randomly sampled from appropriate distributions. Each treated group was compared with the respective biological replicates with no treatment. Therefore, Welch's tests were applied. P‐values were FDR‐corrected according to the Benjamini–Hochberg (BH) method.^[^ ^69^ ^]^ Secreted proteins with significant (FDR < 0.1) absolute log_2_(FC) ≥1 were identified as hits.

### HUVEC Spheroid Sprouting Assay

Spheroids were created with 1000 cells/spheroid using a hanging drop method. The HUVECs were suspended in a spheroid preparation medium (20% of methyl cellulose solution, 70% EGM‐2, and 10% FCS) and pipetted in 25 µL droplets on the inside of the lid of a petri‐dish. One lid was seeded with 120 drops containing HUVECs, which were inverted and placed over a petri dish containing PBS for the spheroids to form overnight. After 24 h, drops were gently harvested, washed (PBS) and 240 spheroids (the content of two lids for two 96 well plates) were transferred into cooled 15 mL falcon tubes. Spheroids were resuspended with 1666 µL rat collagen hydrogel solution. Hydrogel gel solution for two 96 well plates consisted of 133 µL 10x M199 (ThermoScientific), 307 µL ultrapure water (MilliQ, >18MΩ cm), 93 µL NaHCO_3_ 7.5%, 333 µL EGM‐2, and 800 µL 5 mg mL^−1^ Collagen I (rat, #50 201, ibidi GmbH). HUVEC spheroid suspensions were placed in volumes of 8 µL into each well of a “µ‐slides Angiogenesis” 96 well plate (ibidi GmbH). After gelation of the collagen gels (37 °C, 30 min), the embedded spheroids were exposed to either 70 µL EGM‐2 containing 20 ng mL^−1^ bFGF (PeproTech) (pos. control) or CM and incubated for 48 h (37 °C, 5% CO_2_). Spheroids were fixed in Histofix (Roth), and bright‐field images were taken (Zeiss Primovert). A total of 8–38 spheroids per treatment were measured.

### CAM Model

Fertilized chicken eggs (Arare, Switzerland) were incubated in a hatching incubator (relative humidity 65%, 37 °C). Visualization of the CAM vasculature was performed under an epifluorescence microscope (Eclipse E 600 FN; Nikon AG) as previously described.^[^ ^70^ ^]^ On embryo development day (EDD) 7, the plastic sterilized rings (5 mm diameter) were placed on the surface of the CAM and 40 µL of CM were placed into the rings. After 24 h, the same treatments were repeated. Visualization of blood vessels was performed 48 h after the first treatment (EDD 9) through fluorescence angiography after intravenous injection of fluoresceinisothiocyanate dextran (FITC‐dextran, 20 kD, 20 µL, 25 mg mL^−1^). Additionally, 20 µL of India ink (Pelikan, Switzerland) was administered in the embryonic cavity to enhance vascular contrast.

### qPCR

Exposed CAM sections were excised, preserved, and lysed in RLT plus buffer (Qiagen), subsequently disrupted with a tissue homogenizer (VWR), followed by isolation of total RNA (RNeasy mini kit; Qiagen). Sample concentration and purity were measured with NanoDrop One (Isogen) and complementary DNA synthesis (iScript; Bio‐Rad) was performed with 500–1000 ng of input RNA. qPCR (SYBR green; Bio‐Rad) was performed according to manufacturer's instructions and as previously described.^[^ ^51^ ^]^ Assays were run on a Bio‐Rad CFX96 thermal cycler and analyzed using CFX manager software v3.1. Melting curve analysis was performed to verify correct product formation, and samples were excluded if appropriate. Relative gene expression was calculated with the 2ˆ‐dCt method, relative to the average expression of cyclophilin A (peptidylprolyl isomerase A; PPIA) and beta‐actin (ACTB) and expressed as a percentage of control conditions where indicated. Primers are listed in Table S1 (Supporting Information).

### Neurodevelopmental Assays

Neurospheres generated from human fetal neural progenitor cells (hNPCs) were used to assess the impact of CM on proliferation rate, migratory capacity of hNPCs as well as their differentiation potential after plating the neurospheres onto poly‐D‐lysine/laminin‐coated matrices^[^ ^71^ ^]^ (see Supporting Information for extended M&M).

### Statistics

Data were presented as mean (± SEM) of at least three independent experiments (unless stated otherwise) and analyzed using the commercially available GraphPad Prism software (GraphPad Prism 9, GraphPad Software Inc., USA) or “R” (version 4.1.1). Unpaired *t*‐tests or ANOVA (one‐way ANOVA with Dunnett's multiple comparisons correction) were performed.

## Conflict of Interest

The authors declare no conflict of interest.

## Supporting information

Supporting Information

## Data Availability

The data that support the findings of this study are openly available in Zenodo at https://doi.org/10.5281/zenodo.10569874, reference number 10569874.
