# Supplementary material for: Nanoparticles Dysregulate the Human Placental Secretome with Consequences on Angiogenesis and Vascularization
Source: Adv Sci (Weinh). 2024 May 20;11(28):2401060. doi: 10.1002/advs.202401060 (PMC11267331; doi:10.1002/advs.202401060)
Supplement: Supplementary file 1 — Supporting Information [file ADVS-11-2401060-s001.docx]

**Supplementary Information**

**Nanoparticles Dysregulate the Human Placental Secretome with Consequences on Angiogenesis and Vascularization**

Battuja Dugershaw-Kurzer^1,2^, Jonas Bossart^1,2,3^, Marija Buljan^1,3^, Yvette Hannig^1^, Sarah Zehnder^1^, Govind S. Gupta^1^, Vera M. Kissling^1^, Patrycja Nowak-Sliwinska^4,5^, Judy R. van Beijnum^6^, Arjan W. Griffioen^6^, Stefan Masjosthusmann^7^, Etta Zühr^7^, Ellen Fritsche^7,8,9^, René Hornung^10^, Thomas Rduch^1,10^,Tina Buerki-Thurnherr^1*^

^1^ Laboratory for Particles-Biology Interactions, Swiss Federal Laboratories for Materials Science and Technology (Empa), St. Gallen, Switzerland

^2^ Department of Health Sciences and Technology, ETH Zurich, Zurich, Switzerland

^3^ SIB, Swiss Institute of Bioinformatics, Lausanne, Switzerland

^4^ Institute of Pharmaceutical Sciences of Western Switzerland, Geneva, Switzerland

^5^ School of Pharmaceutical Sciences, University of Geneva, Geneva, Switzerland

^6^Angiogenesis Laboratory, Department of Medical Oncology, Amsterdam UMC loacation Vrije Universiteit Amsterdam, Amsterdam, The Netherlands

^7^ IUF - Leibniz Research Institute for Environmental Medicine, Duesseldorf, Germany

^8^ Medical Faculty, Heinrich Heine University, Duesseldorf, Germany

^9^ DNTOX GmbH, Duesseldorf, Germany

^10^ Department of Gynaecology and Obstetrics, Kantonsspital St.Gallen, St. Gallen, Switzerland

* Corresponding author: Tina Buerki-Thurnherr: tina.buerki@empa.ch, Empa, Lerchenfeldstrasse 5, 9014 St. Gallen, Switzerland

**Supplementary Methods**

## NP characterization

***Transmission electron microscopy (TEM).*** For morphology and size analysis of the particles with TEM, 5 µL of a 10 µg/mL SiO_2_, 10 µg/mL TiO_2_ or 4.5 µg/mL DEPs particle suspension in ddH_2_O was drop-casted and air-dried on a carbon-coated grid (C200Cu100, 200 mesh copper, EM Resolutions) that was pre-incubated for 5 min with 0.1 % (w/v) poly-L-lysine solution in H_2_O. The grids were imaged using a Zeiss EM 900 microscope (Carl Zeiss Microscopy GmbH, Germany) at 80 kV and different magnifications. Mean diameter was measured for 48 SiO_2_ particles, 9 TiO_2_ particles inside agglomerates (limited visibility of individual particles inside the agglomerates) and 90 DEPs particles along their longest axis using the TEM user interface iTEM 5.1 software (Build 1700, Olympus), and statistically analyzed using GraphPad Prism.

***Zeta potential.*** The NP suspensions were prepared by gradual dilution in ultrapure water (MilliQ, >18 MΩ cm) supplemented with 10% TM to desired concentrations (0.1 mg/mL TiO2 and SiO2 NPs; 4.5 μg/mL DEPs), and zeta potential measurements were performed immediately after preparation in disposable folded capillary cells (DTS1070, Malvern), using a Zetasizer Nano ZS equipped with a 4.0 mW He-Ne laser operating at 632.8 nm and with an avalanche photodiode detector (Malvern). Equilibration time was set at 180 s and five consecutive measurements were performed (with at least 10 runs and maximum 100 runs; automatic setting of acquisitions for each set).

***Hydrodynamic diameter****.* For hydrodynamic diameter measurements, NP suspensions were prepared in TM (0.1 mg/mL TiO2 and SiO2 NPs; 4.5 μg/mL DEPs) and measured immediately after preparation of the suspensions in disposable PMMA cuvettes (VWR) using the Zetasizer system (equilibration time set at 180 s; five consecutive measurements with 12 runs).

***Limulus amebocyte lysate assay****.* Two formats of the LAL assay, i.e., Pierce™ LAL Chromogenic Endotoxin Quantitation assay (sensitivity 0.1 EU/mL; Thermo Fisher Scientific, MA, USA) and Endosafe R-PTS™ assay were used. The Pierce LAL Chromogenic Endotoxin Quantitation Kit was performed according to the manufacturer’s guidelines for TiO2 and SiO2 NP suspensions (100 μg/mL) and DEPs suspensions (0.45, 4.5 and 45 μg/mL). Test results were valid if three criteria were fulfilled: a spike recovery within a range of 50 ± 200%, a sample coefficient of variation below 25%, and a spike coefficient of variation below 25%.

**NP interference with biological assays**

NPs can interfere with assays (autofluorescence, signal quenching etc.) or absorb molecules/proteins on their surface. Therefore, we have performed interference assays, in particular with those assays where the NPs are not removed (MTS assay). While the presence of DEPs induced an increase in the optical density at concentrations > 10 μg/mL^1^, SiO_2_ and TiO_2_ NPs did not elicit an intrinsic OD signal or interfere with the MTS or Formazan detection at concentrations up to 200 ug/mL (data not shown). However, for most endpoint analyses (e.g. PARP assay, ELISA, secretomics, all indirect exposure analyses), the collected media were centrifuged to remove the NPs, which should minimize NP interference with the assays. For verification, we have performed an interference assay for the hCG ELISA according to a protocol we have previously publishedd^2^. There was no interference due to intrinsic catalytic activity, no OD generation by the NPs and no binding to the antibody or antigen at concentrations up to 100 ug/mL (data not shown). Finally, there is a possibility that NPs could absorb secreted proteins from the conditioned media during the centrifugation step to remove the NPs, which was addressed by performing an hCG ELISA and IL-1β ELISA (Human IL-1 beta/IL-1F2 DuoSet ELISA, DY201, R&D Systems) from NP samples (0.45 μg/mL DEPs, 0.45 μg/mL TiO2 and SiO_2_ NPs) that were spiked with a known amount of hCG (5000 pg/mL) or IL-1β (200 pg/mL) for 1 h (37°C, 5% CO_2_) and centrifuged (25'000 g, 4°C, 20 min). Both, NP-depleted supernatant and the resuspended pellet fraction (1 mL TM) were analyzed. hCG or IL-1β did not decrease in the supernatants or increase in the pellet fraction after the centrifugation, which corroborates that absorption is negligible (possibly due to a rapid protein corona formation in the BSA-containing PM that may prevent non-specific absorption of other proteins) (Fig. S3).

## Tissue lysis and protein content

After exposure to NPs, placental explants were placed in RIPA lysis buffer (Pierce) containing a 1-fold concentration of Mini Protease Inhibitor Cocktail (Roche) and homogenized with a Tissue Ruptor (Quiagen) for 1 min on ice. Samples were further incubated on ice for 30 min followed by centrifugation at 20'000 g (20 min, 4°C) and collection of lysates in fresh microtubes. Whole protein content of placental explants was quantified using the Pierce BCA Protein Assay Kit (Thermofisher, #23225) according to the manufacturer's instructions.

## BeWo trophoblast culture and viability

BeWo b30 cells were provided by Prof. Dr. Ursula Graf-Hausner (Zurich University of Applied Sciences, Wädenswil, Zürich) with permission from Dr. Alan L. Schwartz (Washington University School of Medicine, MO, USA). The cells were cultured in Ham's F-12K medium (Gibco) supplemented with 10% FBS, 1% penicillin/streptomycin and 2 mM L-Glutamine in a humidified incubator at 37 °C with 5% CO_2_ and sub-cultured twice a week. To determine cell viability, 1 x 10^4^ BeWo cells were seeded into each well of a 96-well plate and cultivated overnight at 37 °C / 5% CO_2_. Cells were exposed to particles (0-100 µg/mL for SiO_2_ and TiO_2_ NPs; 0-10 µg/mL DEPs) for 6, 24 and 48 h or positive control cadmium sulphate (CdSO_4_; 1 mM). The MTS assay, which measures mitochondrial activity as an indicator for cell viability, was performed using the CellTiter96 Aqueous One solution (Promega) according to the manufacturer's instructions. Optical density (OD) was measured at 490 nm with a microplate reader (Mithras2 Plate reader, Berthold Technologies) after incubating the cells with the MTS reagent at standard cell culture conditions for 60 min. OD values were blank-corrected and normalized to untreated controls. Interference controls were performed and only DEPs induced non-specific absorbance at concentrations higher than 10 µg/mL.

## HUVEC culture and viability

Human umbilical vascular endothelial cells (HUVECs; LonzaC2519AS; pooled-donor) were cultured in endothelial growth medium-2 (EGM-2, Lonza). 7'500 cells per well were seeded into an opaque 96 well plate (white; PerkinElmer, 6005680). After 4 h of cultivation, HUVECs adhered onto the plastic surface and EGM-2 was carefully aspirated. The cells were covered with 200 µL of CM from 1^st^ trimester and term placental tissue cultures as indicated. The CM were diluted with 20% fresh EGM-2 to avoid toxicity from depletion of nutrients from the 48 h pre-cultivation with the explants. The metabolic activity of HUVECs was measured using non-lytic RealTime-Glo MT cell viability assay (Promega) after 0, 1, 24 and 48 h. 1% Triton X-100 served as the positive control and TM supplemented with 20% EGM-2 as the negatigve control. The assay was performed according to manufacturer's instructions. In brief, “MT cell viability substrate” and “NanoLuc luciferase” were added to the culture medium. Reduction of the "MT cell viability substrate" to a luminescent "NanoLuc Substrate" by viable cells was measured using the GloMax Explorer (Promega) and reported as relative luminescence units (RLU).

## hNPCs cultivation and experimental design of neurodevelopmental toxicity studies

Primary human neural progenitor cells (hNPCs) isolated from cortices of gestational week 16 (GW16) fetuses were purchased from Lonza Verviers SPRL, Belgium (#PT-2599). hNPCs from maximally two individuals (hNPC donors) were used to minimize the biological variability of the conducted experiments. hNPCs were maintained as free-floating neurospheres in proliferation medium consisting of DMEM (#31966-021, Thermo Fisher) and Ham’s F12 (#31765-027, Thermo Fisher) in a 2:1 ratio (v:v) supplemented with 2% B27 (#17504044, Thermo Fisher, United States), 20 ng/mL EGF (#PHG0313, Thermo Fisher), 20 ng/mL basic FGF (bFGF, #233-FB, R&D Systems), and 100 U/mL penicillin and 100 μg/mL streptomycin (#P06-07100, Pan-Biotech). Neurospheres were cultivated at 37 °C and with 5% CO_2_ under standard cell culture conditions in 10 cm diameter cell culture dishes coated with 30 mg/mL poly-2-hydroxyethyl methacrylate (poly-Hema; #P3932, Merck). Spheres were mechanically passaged once a week (2-3 days before the start of the experiment) into cubicles of 0.2 mm edge lengths using a McIlwain tissue chopper (#TC752, Campden Instruments) and half of the culture medium was replaced thrice a week. Neurospheres with a defined size of 0.3 mm were selected for experiments on hNPC proliferation as well as neuronal and astrocyte differentiation and migration (Schematic 1).


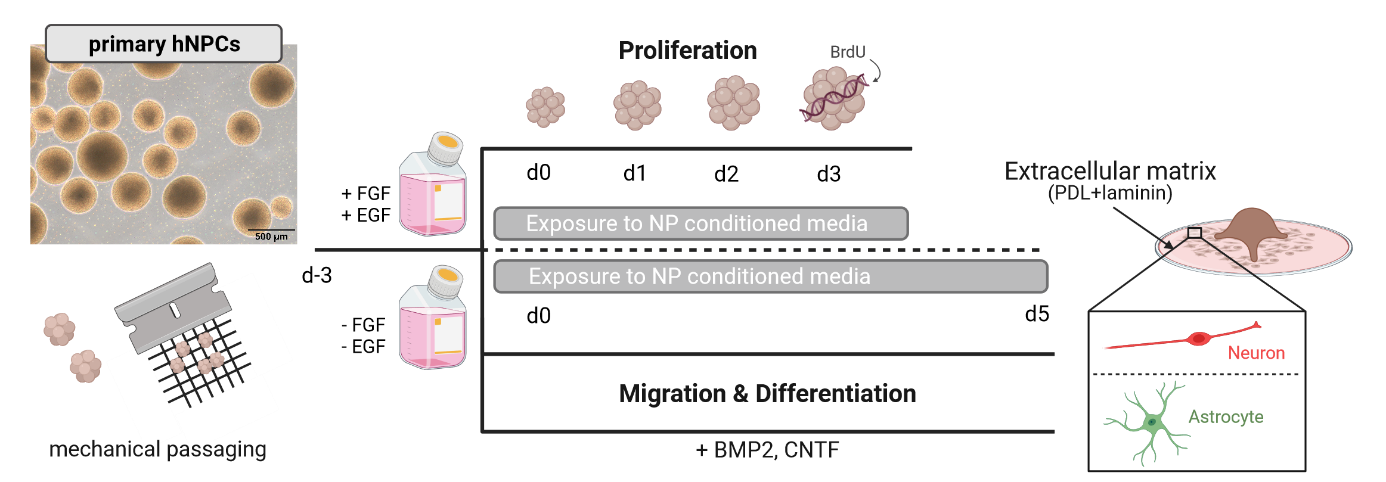


**Schematic 1: Neurosphere experimental setup.** Overview of the study design to assess the impact of CM on neurosphere proliferation, migratory capacity of hNPCs and their differentiation potential into neurons and astrocytes.

***Assessment of hNPC proliferation.*** Proliferation of neurospheres was evaluated by assessing the incorporation of bromodeoxyuridine (BrdU, NPC1b) into the DNA using a commercially available ELISA kit (#11669915001, Roche) according to the manufacturer’s instructions. Therefore, proliferating hNPCs with a diameter of 0.3 mm were used and cultivated for three days in a proliferation medium (BM containing 2% B27 supplement, 20 ng/mL EGF and 20 ng/mL bFGF) or the respective CM supplemented with the same additives. One hNPC neurosphere was cultivated in 100 µL of the respective medium per well of a 96-well plate (U-bottom) coated with 30 mg/mL poly-Hema (poly-Hema; #P3932, Merck) and at least 3-4 technical replicates were prepared for each experimental condition. As an endpoint-specific positive control for reduced hNPC proliferation, hNPCs were cultured in B27 medium without growth factors. In parallel to the evaluation of proliferation, cell viability was assessed after 72 h to discriminate specific effects from unspecific effects originating from decreased cell viability. After 3 days (proliferation) or 5 days (differentiation) of exposure to the CM, mitochondrial activity was assessed using the Alamar blue assay (CellTiter-Blue Assay (CTB), #G8081, Promega).

***Differentiation of hNPCs and immunocytochemical stainings****.* Cell migration and differentiation was initiated by plating 0.3 mm neurospheres into a 96-well plate (F-bottom) coated with 0.1 mg/mL poly-D-lysine (PDL, #P0899-50MG, Merck) and 12.5 μg/mL laminin (#L2020-1MG, Merck) in 100 µL of the CMs or standard hNPC differentiation medium composed of BM supplemented with 1% N2 (#17502-048, Thermo Fisher) and 100U/mL penicillin and 100 μg/mL streptomycin. For untargeted differentiation into a mixed culture of neurons, astrocytes and oligodendrocytes, the respective CM samples were supplemented with 1% N2 supplement (x100). After 5 days of differentiation, the cells were fixated with 4% paraformaldehyde for 30 min at 37 °C and stained with an antibody against β(III)tubulin. Briefly, unspecific binding sides on the fixated cells were blocked with 10% goat serum (GS, #G9023-10ml, Merck) in PBS for 30 min at 37°C. The primary antibody against β(III)tubulin (1:400, rabbit anti-β(III)tubulin monoclonal antibody [EP1569Y]-Alexa Fluor® 647, #ab190575, Abcam, United Kingdom) was incubated overnight in PBS containing 0.01% Triton-X (#T8787, Merck) and 2% GS at 4°C. Following three washing steps with PBS, the cells were incubated with Hoechst34580 (1:100, #H21486, Thermo Fisher) in PBS containing 2% GS for 60 min at 37°C.

The differentiation and maturation of astrocytes from hNPCs was enhanced by the addition of 50 ng/mL BMP2 (#355-BM-010/CF, R&D Systems) and 50 ng/mL CNTF (#257-NT-010/CF, R&D Systems) to all CM and media control (BM with 1% N2) conditions during the 5 days of differentiation. To confirm that the assay setup can detect the inflammatory activation of astrocytes, after 4 days of differentiation, hNPCs differentiating in standard astrocyte differentiation medium were exposed to 10 ng/mL TNF-α (#210-TA-020/CF, R&D Systems) for 24 h. After a total differentiation time of 5 days, astrocytes were fixated with 4% paraformaldehyde for 30 min at 37°C and stained with antibodies against GFAP (1:250, rabbit anti-GFAP monoclonal antibody [EPR1034Y], #ab68428, Abcam) or ICAM-1 (1:50, mouse anti-ICAM-1/CD54 monoclonal antibody, #BBA3 R&D Systems). Therefore, unspecific binding sides were blocked with 10% goat serum in PBS for 30 min at 37 °C. Primary antibodies against GFAP (1:250) or ICAM-1 (1:50) were diluted in PBS containing 0.01% Triton-X and 2% GS and incubated overnight at 4 °C. Cells were washed three times with PBS and then incubated with the respective secondary antibody for the GFAP-staining (1:400, goat anti-rabbit IgG Alexa Fluor™ 488, #A-11008, Thermo Fisher) or ICAM-1-staining (1:400, goat anti-mouse IgG Alexa Fluor™ 546, #A-11030, Thermo Fisher) and Hoechst34580 (1:100, #H21486, Thermo Fisher) in PBS containing 2% GS for 60 min at 37°C. Images of immunocytochemical stainings were acquired and evaluated with the High Content Screening Platform CellInsight CX7 (software version 6.6.3; Thermo Fisher Scientific).The high-content analysis (HCA) tool Omnisphero was used^3^ to stich the individually acquired fields of one well into a single coherent image.

***Quantification of astrocyte and neuronal migration and differentiation.*** After plating hNPC-based neurospheres on PDL-laminin coated 96-well plates, neural progenitor cells start to radially migrate out of the sphere core, form a circular migration area and differentiate into the main effector cells (neurons, astrocytes and oligodendrocytes). In cultures enriched for astrocytes, using BMP2 and CNTF, migration of astrocytes was assessed manually on day 5 of differentiation using bright-field microscopy as previously described by^4^. Since the treatment with BMP2 and CNTF results in almost pure astrocyte cultures, information about astrocyte migration can be obtained by analyzing bright-field images. In spontaneously differentiated cultures, cell migration of differentiating neural progenitor was evaluated automatically by analysing the area of Hoechst34580-stained nuclei with the Omnisphero software according to^3^ and as previously described by^4^. Cell migration is thereby evaluated by defining the area of Hoechst34580-stained nuclei. Quantification of neurons and astrocytes was performed using the HCS Studio™ Cell Analysis Software provided by Thermo Fisher. The number of valid neuronal nuclei in the well was determined using the Neuronal Profiling V4 BioApplication Software. Therefore, valid neuronal cells were quantified by combined analysis of fluorescence channel 1 (Hoechst34580) identifying the cell nuclei and channel 2 (β(III)tubulin, Alexa647®) identifying neuronal bodies. Following background correction, an independent fixed intensity threshold was set for each channel to ensure proper quantitative analysis. Nucleus and cell body features were computed and independently assigned or excluded based on pre-defined object selection parameters of the respective channel. Border-touching objects were excluded on individual image/field levels. Neuronal differentiation is depicted as the number of cells positively stained for β(III)tubulin as a percentage of the total nuclei count within the migration area for each technical replicate (well). Astrocytes were quantified using the Colocalization V4 BioApplication Software. After background correction, object selection parameters (intensity, object area and shape) were set for each channel (nuclei (Hoechst34580), astrocytes (Alexa488®)). Border-touching objects were excluded on individual image/field level. Object identification was performed on the basis of a fixed intensity threshold independently set for each channel. The number of astrocytes is defined by the number of nuclei colocalized with a GFAP-positive cell body as a percentage of the total nuclei count within the migration area of one technical replicate (well).

**Supplementary figures**


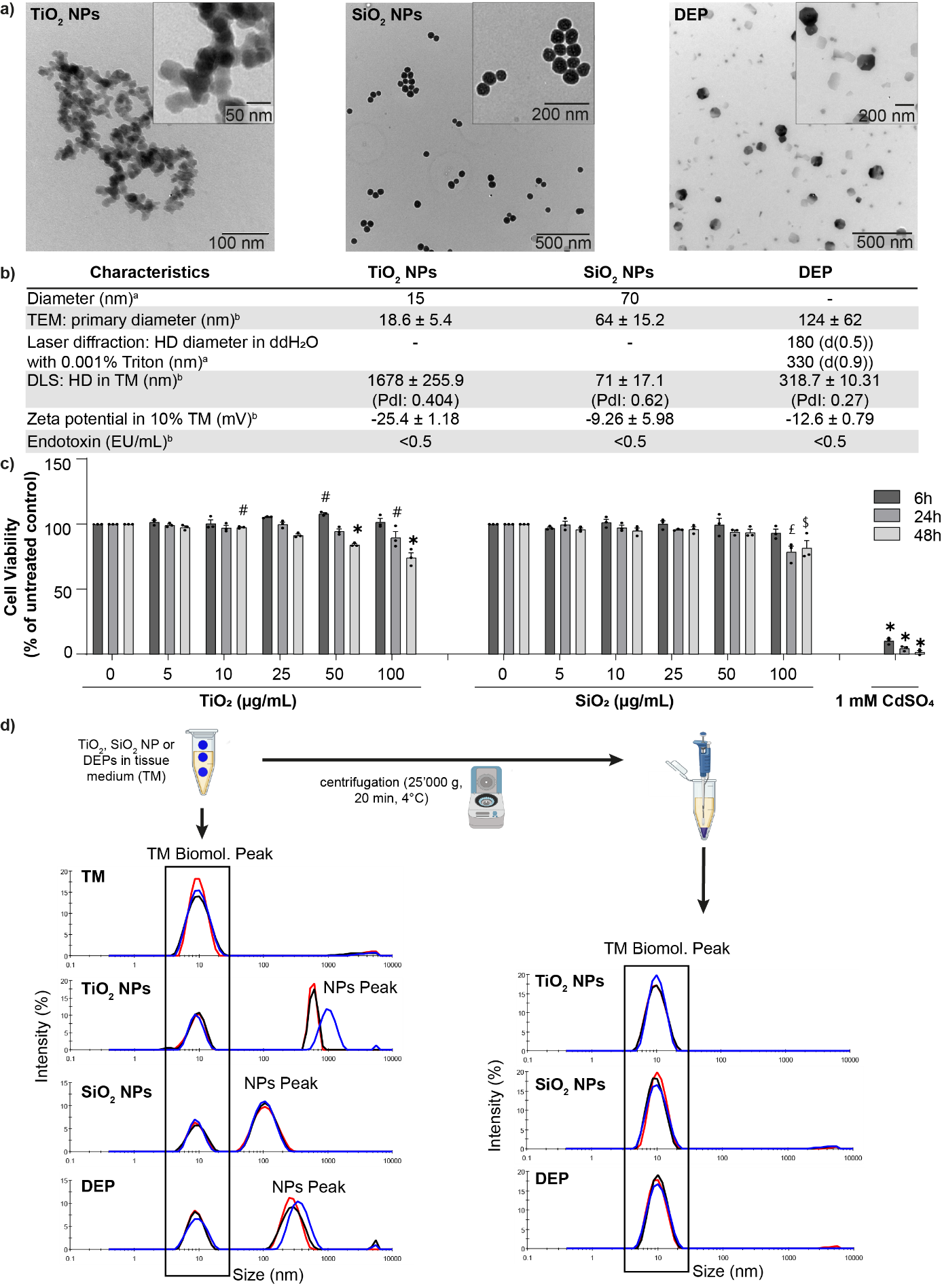


**Fig. S1:** **NP characterization, dose-response establishment in BeWo b30 cells and NP removal from CM.** a) TEM images of TiO_2_, SiO_2_ NPs and DEPs. b) Summary of physicochemical properties. Abbreviations: DLS: dynamic light scattering; d(0.5): mean particle-size indicating the diameter below which 50% of the volume is present; d(0.9): mean particle-size indicating the diameter below which 90% of the volume is present; HD: hydrodynamic diameter; PdI: polydispersity index; TM: tissue medium. a: data supplied by the manufacturer; b: experimentally determined. c) Cell viability of BeWo b30 trophoblast cells after exposure to TiO_2_, SiO_2_ and DEPs for 6, 24, and 48 h determined by MTS assay. 1 mM CdSO_4_ was applied as a positive control. Data represent the mean (± SEM) percentages of viable cells compared to the untreated control of three independent biological experiments with three technical repeats each. One-way ANOVA with Dunnett's multiple comparisons correction was used for the analysis of comparisons between control and the treatments (left to right: #p = 0.0145, 0.0214, 0.03080; $p = 0.0025, £p = 0.0001, *p < 0.0001). d) DLS measurements confirming the removal of NPs from TiO_2_ and SiO_2_ NP (25 µg/mL) or DEPs (0.45 µg/mL) suspensions by centrifugation. The presented data is from three independent readings (Peaks: Red - read 1, Black - read 2, Blue - read 3).


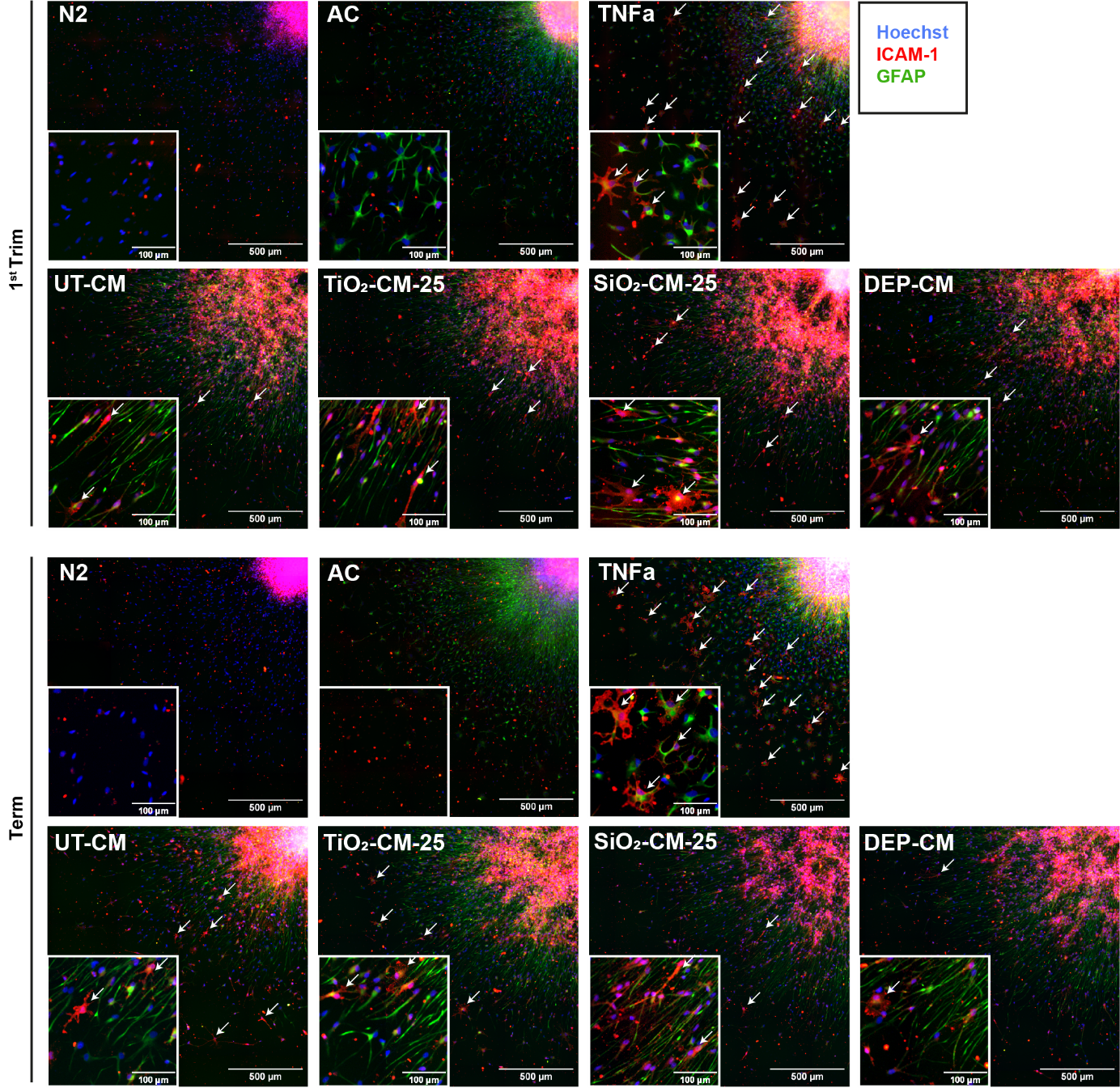


**Fig. S2:** **Impact of CM from first trimester and term placental explants exposed to TiO_2_, SiO_2_ NPs or DEPs on astrocyte differentiation and inflammatory activation.** Immunofluorescent staining of ICAM-1+ astrocytes after exposure of differentiating astrocytes with CM from NP-exposed explants or control media for 5 days. White arrows highlight ICAM-1+ cells in the migration area. 10 ng/mL TNF-α was used as a positive control for astrocyte inflammatory activation. CM samples from individual placenta donors were defined as independent biological replicates. Representative fluorescent images from at least four independent experiments per condition (1^st^ trim: n = 5, term: n = 4). N2: differentiation control without AC-enhancing factors, AC: astrocyte differentiation control.


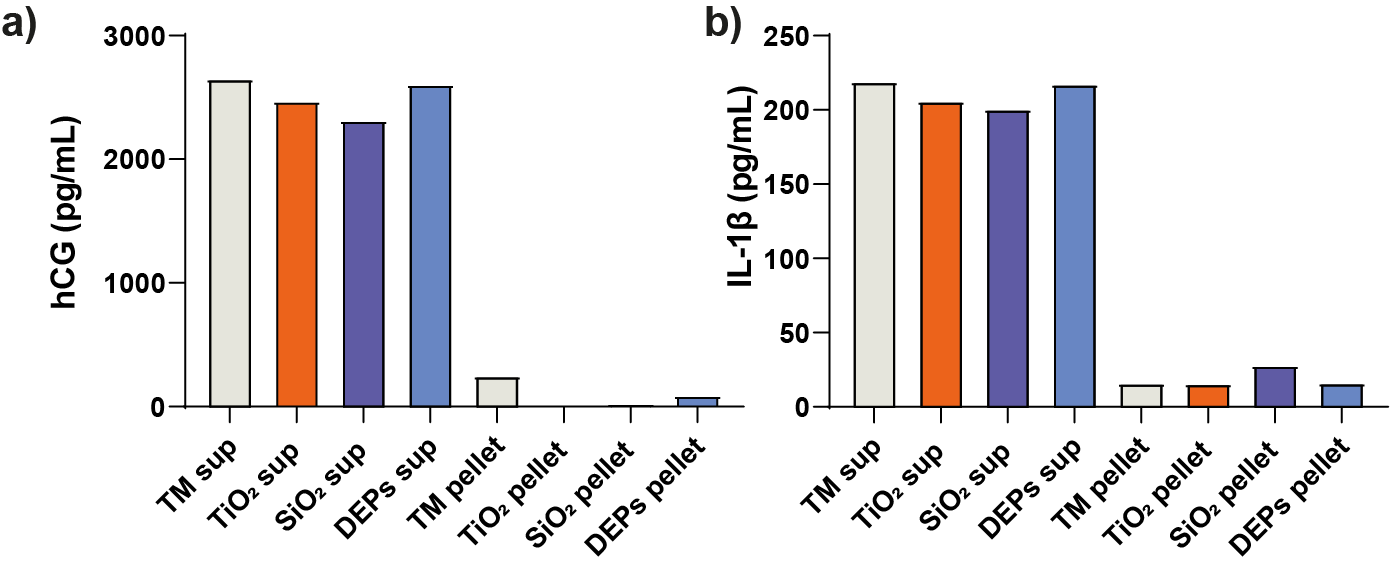


**Fig. S3:** **Adsorption of placental mediators to TiO_2_, SiO_2_ NPs or DEPs.** A known amount of hCG (2000 pg/mL) or IL-1β (200 pg/mL) was incubated with particles (0.45 μg/mL DEPs, 0.45 μg/mL TiO_2_ and SiO_2_ NPs) for 1 h (37°C, 5% CO_2_) and centrifuged (25'000 g, 4°C, 20 min) to pellet the NPs. Both, NP-depleted supernatants (sup) and the resuspended pellet fraction were subjected to hCG (a) and IL-1β ELISA (b) to assess potential depletion of the proteins from the media. Data are from one experiment.

**Supplementary tables**

**Table S1:** List of primer sequences.

| Oligo Name | Forward primer | Reverse primer |
| --- | --- | --- |
| PPIA | AAGGAGGGGATGAACGTG | AGCTGCCCGCAGTTGGA |
| CD31 | ACTCCAGTGGCATGAAAACT | ACAGAGCAGAGAAAAGTGGTC |
| Itgav | AAACACTCATGACTGCAAGGA | TCAGTGCAGACGTGTTAGTCAC |
| itgb3 | AGACAGCTACGTTGGTGATGAA | CAGCATCCTACATGTTCCCA |
| ACTB | AGACAGCTACGTTGGTGATGAA | TGCTCCTCAGGGGCTACTCT |
| VEGFA | GACCTGTAAATGTTCCTGCAA | AGAAATCAGGCTCCAGAAACA |
| PDGFRA | ATCTCAGCGTTGTTTGCCA | AATTCTATCCATCAAAGTCATAGG |
| PDGFRB | AAGGATAGGCGATGTCATGG | ATACCTGTCAGAGTGAGTGGGG |

**Supplementary references**

1. Bongaerts, E., et al., *Label-free detection of uptake, accumulation, and translocation of diesel exhaust particles in ex vivo perfused human placenta.* J Nanobiotechnology, **2021**. 19(1): p. 144.

2. Drasler, B., et al., *Single exposure to aerosolized graphene oxide and graphene nanoplatelets did not initiate an acute biological response in a 3D human lung model.* Carbon, **2018**. 137: p. 125-135.

3. Schmuck, M.R., et al., *Omnisphero: a high-content image analysis (HCA) approach for phenotypic developmental neurotoxicity (DNT) screenings of organoid neurosphere cultures in vitro.* Arch Toxicol, **2017**. 91(4): p. 2017-2028.

4. Koch, K., et al., *Scientific Validation of Human Neurosphere Assays for Developmental Neurotoxicity Evaluation.* Front Toxicol, **2022**. 4: p. 816370.
